# Systematic literature review and meta-analysis of health state utility values in metastatic castration-resistant prostate cancer

**DOI:** 10.1093/oncolo/oyae321

**Published:** 2024-11-26

**Authors:** Elena Castro, Rhett Figliuzzi, Sarah Walsh, Samantha Craigie, Jonathan Nazari, Alexander Niyazov, Imtiaz A Samjoo

**Affiliations:** Hospital Universitario 12 de Octubre, Unidad C. Cáncer Genitourinario, Sistema Nervioso Central, Sarcomas y Tumores Cutáneos, 28041 Madrid, Spain; EVERSANA, Value & Evidence, Burlington, ON L7N 3H8, Canada; EVERSANA, Value & Evidence, Burlington, ON L7N 3H8, Canada; EVERSANA, Value & Evidence, Burlington, ON L7N 3H8, Canada; Pfizer Inc., New York, New York 10017, United States; Pfizer Inc., New York, New York 10017, United States; EVERSANA, Value & Evidence, Burlington, ON L7N 3H8, Canada

**Keywords:** metastatic castration-resistant prostate cancer, health state utility values, systematic literature review, meta-analysis

## Abstract

Despite being an important goal, the preservation of quality of life of patients with metastatic castration-resistant prostate cancer (mCRPC) is poorly characterized across lines of therapy. In this review, a systematic literature review and meta-analysis were conducted to synthesize EuroQoL 5-Dimension (EQ-5D) data among adult men with asymptomatic or mildly symptomatic mCRPC in both first line (1L) and second line and later (2L+) therapy. MEDLINE, Embase, and Cochrane CENTRAL were searched from inception to October 2022 using Ovid. Supplemental searches of other data sources were also conducted (PROSPERO registration: CRD42021283512). Meta-analyses were conducted to estimate pooled EQ-5D index utility values and EQ visual analog scale (VAS) scores in both 1L and 2L+. Various sensitivity analyses were also conducted. Forty-five unique publications met the inclusion criteria. In primary studies, baseline EQ-5D index utility values ranged from 0.7 to 0.9 in 1L and 0.63 to 0.7 in 2L+. Twelve trials and observational studies were feasible for inclusion in the meta-analysis. The pooled mean baseline EQ-5D index utility value was estimated as 0.79 (95% CI, 0.70–0.84) and 0.69 (95% CI, 0.67–0.71) for 1L (*n* = 7 studies) and 2L + (*n* = 4 studies), respectively. The pooled mean baseline EQ VAS score was estimated as 74.63 (95% CI, 70.97–78.29) and 65.82 (95% CI, 64.53–67.11) in 1L and 2L+, respectively. Limitations include hampered comparability between studies due to heterogeneity in study design and geographical regions. This study provides a comprehensive synthesis of EQ-5D data presently available in adults with mCRPC in both 1L and 2L + therapy.

**Implications for practice**

The preservation of quality of life (QoL) (such as measured by EuroQoL 5-Dimension) in patients with metastatic castration-resistant prostate cancer (mCRPC) remains a goal as important as the extension of life, particularly in advanced stages of the disease. The results of these analyses can act as a valuable resource for those developing cost-utility analyses and other analyses related to QoL, be used to help inform or validate utility value inputs, and to support drug reimbursement in mCRPC and highlight the need for more standardized QoL reporting.

## Introduction

Prostate cancer (PC) is the third most diagnosed malignancy according to the World Health Organization and accounts for 7% of newly diagnosed cancers in men globally.^1,2^ As the disease progresses, many patients will develop an advanced form of the disease called metastatic castration-resistant prostate cancer (mCRPC).^3^ Due to the poor prognosis and the incurable nature of mCRPC, the goals of mCRPC therapy involve mitigating progression, prolonging life, and maintaining quality of life (QoL).^4^

Quality of life is an important outcome not only for patients and their caregivers but also in the approval and reimbursement of cancer therapies. QoL can be measured by using utility values, such as the EuroQoL 5-dimension (EQ-5D) family of instruments. EQ-5D measures QoL using 5 dimensions of health—mobility, self-care, usual activities, pain & discomfort, and anxiety & depression.^5^ EQ-5D instruments are recommended for use by many health technology assessment (HTA) bodies.^5^ There are 2 main versions of the EQ-5D questionnaire: a version with 3 response levels (EQ-5D-3L) and a version with 5 response levels (EQ-5D-5L). In both the EQ-5D-3L and -5L scales, the total scores from the responses in the questionnaires are converted into a single index value using country-specific scoring algorithms and are typically used for utility values in cost-utility analyses (CUAs).^6,7^ The EQ visual analog scale (VAS) is included in both the EQ-5D-3L and -5L to provide a score representing a patient’s own judgment of their overall health, measured on a vertical visual scale from “0” (“the worst health you can imagine”) to “100” (“the best health you can imagine”).^6,7^ Both index values and VAS scores are valuable in representing a patient’s QoL; however, only index values are used as health state utility values (HSUVs) to calculate quality-adjusted life years in CUAs.^8^

As new therapies and treatment combinations are developed for mCRPC, utility values and QoL will continue to play a major role in the economic evaluations of these therapies. The importance of QoL in determining the value of an anti-cancer therapy (such as those used in mCRPC) is highlighted by the recognition and inclusion of QoL in the European Society of Medical Oncology’s Magnitude of Clinical Benefit Scale.^9^ There are several challenges in implementing utility values in CUAs: (1) large variations in utility values are often reported in studies, which could bias the conclusions from a CUA; (2) the quality of studies can be variable, and (3) even if a utility value is of high quality and accurate, the context of the utility value may be missing and not be appropriate for use in a specific economic evaluation.^10^ To accurately evaluate these treatments, using appropriate utility values in CUAs will be imperative. The objective of this study was to conduct a systematic literature review (SLR) and meta-analysis to identify and synthesize EQ-5D utility values in the first (1L) and later lines (2L+) of therapy in asymptomatic or mildly symptomatic mCRPC.

## Material and methods

### Systematic literature search

The SLR was designed and reported according to the Preferred Reporting Items for Systematic Literature Reviews and Meta-Analyses (PRISMA) statement^11^ to identify clinical studies (ie, clinical trials and observational studies), economic evaluations, utility elicitation studies, and HTA documents assessing and reporting utility data in patients with mCRPC. The review protocol was developed in accordance with the PRISMA for systematic review protocols statement^11^ and was registered with PROSPERO international prospective register of systematic reviews a priori (registration number CRD42021283512), which outlined the population, intervention, comparator, outcomes, and study design (PICOS) criteria and methodology used to conduct the review (Supplementary Table S1). The Ovid platform was used to search Embase, MEDLINE , Ovid MEDLINE Daily, and the Cochrane Central Register of Controlled Trials by an experienced information specialist in consultation with the review team. The search strategy used a combination of controlled vocabulary (eg, “mCRPC” or “Metastatic castration-resistant prostate cancer”) and keywords (eg, “utility values”). Published and validated filters were used to select for study design and were supplemented using additional medical subject headings terms and keywords to isolate specific instruments.^12^ Gray literature sources such as conferences, HTA and regulatory bodies, and health economic databases were also searched by hand. Further details of the search, including the conferences and databases searched, are provided in the Supplementary Materials A and Tables S2–S4).

### Study selection and data extraction

Study selection was performed using pre-specified PICOS criteria, which are fully detailed in Supplementary Table S1 in the Supplementary Materials. Patients were required to have undergone medical or surgical castration and have asymptomatic or mildly symptomatic mCRPC and could receive any intervention for mCRPC. Study designs included but were not limited to clinical trials, observational studies, economic evaluations, and utility elicitation studies. Included outcomes for the SLR were generic (EQ-5D-5L, EQ-5D-3L, Health Utilities Index 3 [HUI3], Assessment of Quality of Life [AQoL], etc.) or disease-specific (European Organization for Research and Treatment of Cancer Quality of Life Questionnaire – Prostate Cancer Module [EORTC QLQ-PR25], Functional Assessment of Cancer Therapy Prostate [FACT-P], etc.) QoL instruments, as well as values that could be mapped to HSUVs. For the present objective, studies identified and included in the SLR were not included in this analysis if they did not report utility data (ie, EQ-5D, EQ-5D-5L, EQ-5D-3L, Short Form 6-Dimension [SF-6D], HUI3, Quality of Well-Being [QWB] Index, 15D, AQoL). It should also be noted that EQ VAS scores were included as part of the EQ-5D umbrella.

For studies that met the eligibility criteria, data were exacted into standardized tables in Microsoft Excel (Microsoft Corporation). The data extraction form was piloted by extracting selected included studies into the form, which was then refined and used as training for the reviewers to ensure that study and patient characteristics and outcome measures were collected consistently. Studies were extracted by a single reviewer and then independently assessed for accuracy and completeness by a second reviewer. Discrepancies were solved by a third independent reviewer. Data captured from the studies included publication characteristics, study setting, study methods, study participants, and study findings. Further details on study selection and data extraction are available in the Supplementary Materials A.

### Quality assessment

Quality assessment was performed based on the type of study. For utility value evidence reported in primary studies, the quality assessment was performed using the NICE Quality Assessment Checklist for HSUVs.^13^ For economic evaluations, quality assessment was performed using the Drummond and Jefferson Checklist.^14^ Both types of quality assessment were conducted by a single reviewer and validated for consistency and integrity by a second reviewer. Please refer to Appendix A for additional details of the quality assessment (Supplementary Materials A and Tables S6 and S7).

### Feasibility assessment

To ensure sensible and robust comparisons in the meta-analysis, the similarity of included studies was qualitatively assessed. Included studies that reported common outcomes were compared to assess their clinical and methodological similarities. Consistency across relevant studies was evaluated by comparing the utility value instruments and the timepoints they were assessed. For data to be considered sufficiently similar for meta-analysis, studies were required to have utility instruments and timepoints in common, have comparable patient populations (including line of therapy in mCRPC setting), and present utility values with SD or SE. In addition, a rigorous qualitative between-study heterogeneity evaluation for the following elements was conducted: study design, patient characteristics, timepoints assessed, line of therapy at which treatments for mCRPC were received, and prior treatment(s) received. If heterogeneity in these elements was detected, a sensitivity analysis of the variable was conducted if considered feasible.

### Meta-analysis

A meta-analysis was considered feasible if 2 or more studies were sufficiently similar in study characteristics, baseline patient characteristics, measurement of outcomes, and reported endpoints in a specific line of therapy; and reported EQ-5D utility values or EQ VAS scores for mCRPC, complete with SD or SE. A conventional frequentist meta-analysis was conducted in R Version 3.6.1 using meta package Version 4.18-0 to provide mean pooled treatment effect and 95% CIs by line of therapy using fixed-effect and random-effects models according to Cochrane guidelines.^15^ The random-effects model was conducted to account for both variances within studies and variances between studies. Additionally, sensitivity analyses were planned to determine if certain study characteristics disproportionately influence the results of the meta-analysis. Outliers were not considered due to the small number of included studies in the analysis. No assumptions were made for missing data. Heterogeneity was evaluated using the I^2^ and τ^2^ statistic. The primary meta-analysis was performed for all patients, regardless of the study design (ie, clinical trials and observational studies), according to the line of therapy (ie, 1L and 2L+) and instrument used (ie, EQ-5D index value or EQ VAS score). Sensitivity meta-analyses were performed according to the study design and level of instrument used (ie, EQ-5D-3L and EQ-5D-5L, clinical trials, and observational studies). Additional meta-analysis details are provided in the Supplementary Materials A.

## Results

### Literature search

A total of 4 429 records were identified through the database and gray literature searches, of which 185 records (155 publications and 30 HTA documents) met our eligibility criteria. After further exclusion of studies which did not report utility values or VAS, a total of 54 records reporting on 23 primary publications (including 8 unique clinical trials and/or analyses of clinical trials, 9 unique observation studies, 2 unique utility mapping studies, 1 unique utility weight estimation study), 15 unique economic evaluations, and 16 HTA documents (including 10 unique reports containing economic evaluations) fulfilled the inclusion criteria and are the focus of the current paper (Supplementary Figure S1, Table S5). Quality assessment of the included studies found that the reporting of data collection, methods, analysis, and interpretation of results was of moderate quality (Supplementary Tables S6 and S7). Details of the 46 unique records considered, including all utility outcomes, are presented in the Supplementary Materials Tables S8–S10. Due to the scarcity of data on other utility outcomes, only the EQ-5D index utility values and EQ VAS scores are elaborated on in the ensuing sections by study type.

### Summary from primary studies

#### Study characteristics

Overall, 10 unique primary studies were identified in the 1L mCRPC setting (Supplementary Materials A and Table S8), with 4 clinical trials^16–20^ and 6 observational studies.^21–27^ In the 2L + setting, 7 unique studies were identified (Supplementary Materials A and Table S8), with 4 clinical trials,^28–32^ 2 observational studies,^23,33^ and one mapping study.^34^ Five primary studies did not specify the line of therapy evaluated for their utility values or VAS scores presented: 3 were observational studies,^27,35,36^ one was a utility mapping study,^37^ and one was a utility weights study.^38^

#### EQ-5D and VAS data from primary studies by line of therapy

Index utility values and VAS scores at baseline ranged from 0.7 to 0.9^17,19-24,26,27^ and 68.0 to 77.7,^16,17,19,21,23,24,27^ respectively, in the 1L setting and from 0.6 to 0.7^23,28–30,34^ and 62.7 to 68.0,^23,29–31^ respectively, in the 2L + setting (Table 1). When comparing EQ values across lines of therapy, ranges of both baseline EQ-5D index values and EQ VAS scores were notably lower in the 2L + setting vs the 1L setting (Table 1). Four primary studies did not report baseline EQ-5D index values or EQ VAS scores by line of therapy; however, their values overlapped with the ranges reported by studies in the 1L and 2L + settings (Table 1).^27,35–37^

**Table 1.** EQ-5D index utility values and EQ VAS from primary studies by line of therapy.^a^

1L		2L+		Unspecified LoT	
11 unique studies		8 unique studies		4 unique studies	
EQ-5D index (baseline)	EQ VAS (baseline)	EQ-5D index (baseline)	EQ VAS (baseline)	EQ-5D index (baseline)	EQ VAS (baseline)
0.7–0.9^b^	68.0–77.7	0.63–0.7	62.7–68.0	0.635–0.84	53.4–73.9

^a^Utility values from all EQ-5D instruments (ie, EQ-5D-3L, EQ-5D-5L, unspecified EQ-5D) were combined for presentation, as many studies did not specify the EQ-5D scale used. Studies reporting baseline utilities using alternative scales (such as AQoL, QWB, etc.) were not included in this results summary but are reported in the appendix.

^b^This value represents a median baseline EQ-5D index value. Other values in the range were mean baseline EQ-5D index values.

Abbreviations: 1L, first line; 2L+, second line and later; EQ-5D, EuroQoL 5-Dimension; LoT, line of therapy; VAS, visual analog scale.

#### EQ-5D and VAS data from primary studies by treatment

In several studies, EQ-5D index utility values and EQ VAS scores were reported by treatment in the line of therapy of interest. Where data were available for a specific treatment in both 1L and 2L+, a lower EQ-5D index utility value and EQ VAS score was observed in 2L + compared with 1L. For more details, see the Supplementary Materials A and Table S11.

### Summary from economic evaluations

#### Study characteristics

A total of 15 unique economic evaluation studies and 10 unique HTA documents containing economic evaluations fulfilled the eligibility criteria (Supplementary Materials A and Tables S9 and S10).

#### EQ-5D data from economic studies by health state

In short, when comparing between treatment lines (ie, 1L and 2L+), the range of reported HSUVs for a given health state was higher in 1L than 2L+. More detailed results by health state are presented in Supplementary Materials A and Tables S12 and S13.

#### EQ-5D data from economic studies by treatment

Where reported, EQ-5D index utility values for a specific treatment were reported as utility increments or decrements. Details of treatment-specific utilities reported by the included economic evaluation studies are presented in Supplementary Table S14.

### Feasibility assessment results

Heterogeneity was observed in the availability of data in the included studies. The primary studies, Dearden et al and Boye et al, did not report the SD or SE of the utility values presented, and therefore, the data from such studies were deemed insufficient to conduct a meta-analysis. ^24,36^ Similarly, data from economic evaluations and HTA documents were also insufficient for conducting meta-analyses.

Heterogeneity was also observed in the utility instrument reported and the timepoint it was measured. Most studies reported utility data or VAS using a consistent timepoint such as mean baseline EQ-5D index utility values and/or EQ VAS (Supplementary Table S5). However, several studies reported instruments other than EQ-5D or EQ VAS such as AQoL, QWB, and Standard Gamble at varying timepoints.^25,26,32,33,38^ Due to the small sample of studies reporting consistent scales and timepoints for these other utility instruments, a meta-analysis was not considered feasible. Finally, 2 studies, although reporting relevant data, did not describe the line of therapy and were therefore excluded from the meta-analyses.^35,37^

Overall, 14 records reporting on 12 unique studies were deemed sufficiently similar to derive valid pooled estimates of EQ-5D index utility values and EQ VAS scores in a meta-analysis, including 8 unique studies reporting clinical trial data and 4 unique studies reporting observational data. Details of the 12 unique studies included in the meta-analysis are presented in Table 2. Due to the small sample of studies in our evidence base, studies with different designs (eg, clinical trials and observational studies) or EQ-5D scale used (EQ-5D-3L, EQ-5D-5L, and unspecified EQ-5D) were combined for the meta-analysis. To account for this, a sensitivity analysis of clinical trials only and observational studies only was performed in 1L, but not 2L + , due to lack of data. A sensitivity analysis using only EQ-5D-5L index utility values alone was also performed in both 1L and 2L+. A sensitivity analysis of EQ-5D-3L alone could not be performed due to lack of data.

**Table 2.** List of studies included in the meta-analysis.

Reference	Data source	LoT	Treatment	Outcome	Sample size	Mean baseline outcome (SD)
^17^	Clinical trial	1L	Enzalutamide	EQ-5D-5L EQ VAS	184	0.81 (0.2) 77.7 (15.48)
		1L	Bicalutamide		191	0.83 (0.18) 76.9 (17.73)
^18^ ^19^	Clinical trial	1L	Enzalutamide	Unspecified EQ-5D EQ VAS	872	0.85 (0.15) 77.15 (16.72)
		1L	Placebo		845	0.84 (0.17) 75.94 (17.53)
^20^	Clinical trial	1L	Enzalutamide	Unspecified EQ-5D	573	0.829 (0.154)
^21^	Observational	1L	Docetaxel	EQ-5D-5L EQ VAS	50	0.83 (0.126) 77.5 (12.6)
^22^	Observational	1L	Unspecified	EQ-5D-3L	602	0.7 (0.49)^a^
^27^	Observational	1L	Unspecified	Unspecified EQ-5D EQ VAS	112	0.82 (0.16) 72.9 (17)
^16^	Clinical trial	1L	Enzalutamide	EQ VAS	198	74.8 (16)
		1L	Placebo		190	76.6 (15.9)
^23^	Observational	1L	Enzalutamide	EQ-5D-5L EQ VAS	1175	0.71 (0.24) 68 (19)
		2L	Enzalutamide Post-chemo		398	0.68 (0.24) 66.2 (20.4)
		2L	Enzalutamide Post-Abiraterone Acetate		42	0.67 (0.27) 63.6 (20.8)
		3L	Enzalutamide		94	0.63 (0.24) 62.7 (20.1)
^28^	Clinical trial	2L	Cabazitaxel	EQ-5D-3L	100	0.699 (0.044)
^29^ ^30^	Clinical trial	3L	Cabazitaxel	EQ-5D-5L EQ VAS	113	0.7 (0.26) 65.8 (20.4)
		3L	Enzalutamide or Abiraterone acetate		112	0.7 (0.22) 66.3 (18.5)
^34^	Clinical trial	2L+	Unspecified	Unspecified EQ-5D	209	0.688 (0.282)
^31^	Clinical trial	3L	Olaparib + Abiraterone acetate	EQ VAS	71	65 (20.9)
		3L	Placebo + Abiraterone acetate		71	68 (16.5)

^a^SD calculated from SE.

Abbreviations: 1L, first line; 2L, second line, 2L+, second line or later; 3L, third line; EQ-5D, EuroQoL 5-dimension; SD, standard deviation; VAS, visual analog scale.

### Meta-analysis

A meta-analysis was conducted on mean baseline EQ-5D index utility values and EQ VAS based on the results of the feasibility assessment. Supplementary Table S15 presents a summary of meta-analysis results.

#### EQ-5D first line

**Figure 1.**
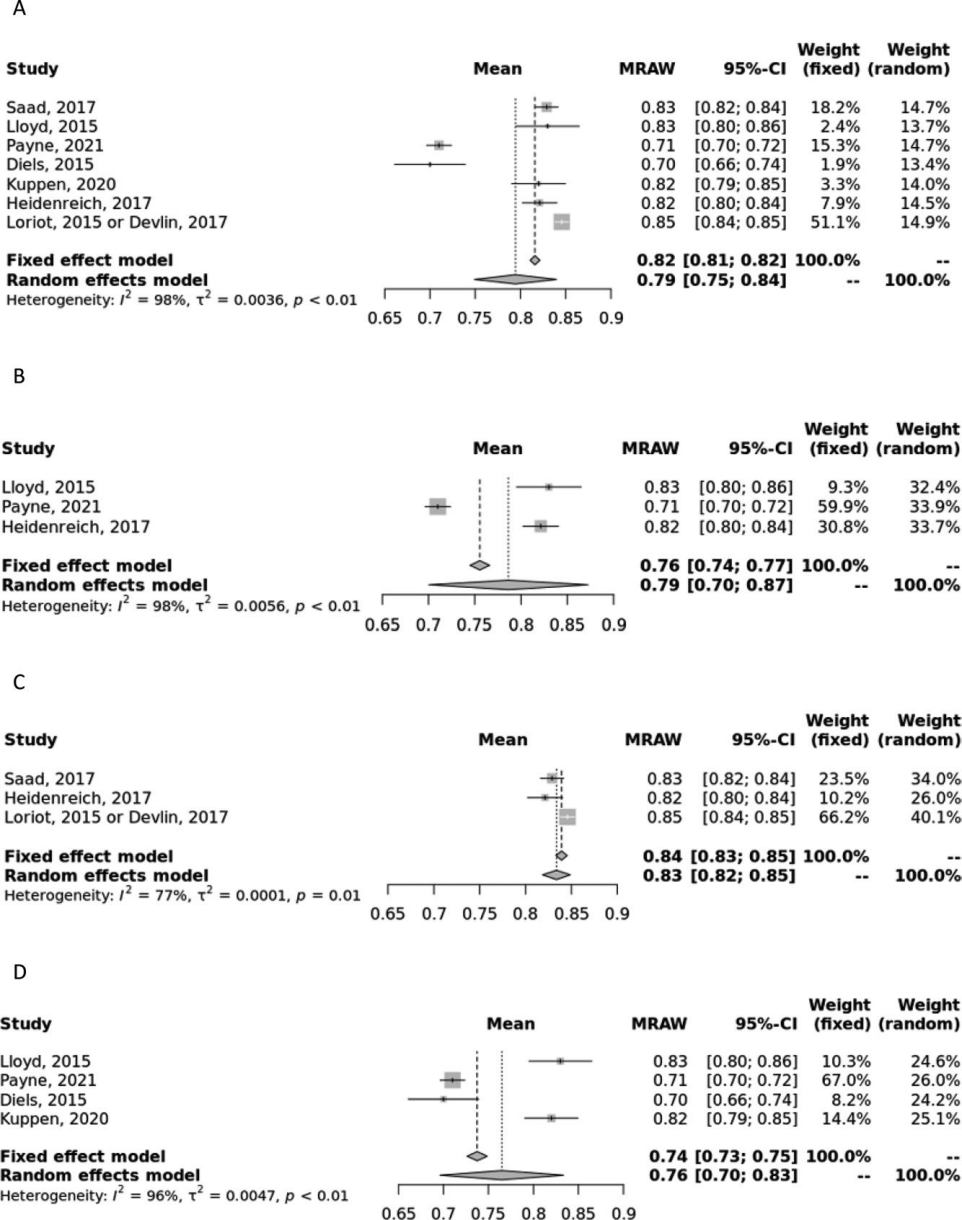
Meta-analysis of first-line EQ-5D index scores. 1. (A) Forest plot of combined first-line EQ-5D-3L and EQ-5D-5L index scores in all primary studies (clinical trials and observational studies). (B) Forest plot of only first-line EQ-5D-5L index scores in all primary studies. (C) Forest plot of combined first-line EQ-5D-3L and EQ-5D-5L index scores from clinical trials only. (D) Forest plot of combined first-line EQ-5D-3L and EQ-5D-5L index scores from observational studies only. Abbreviation: MRAW, raw, untransformed mean.

When all 1L mean baseline EQ-5D index utility values were combined for the primary analysis,^17-23,27^ the pooled estimate for EQ-5D index utility values (95% CI) was 0.79 (0.75, 0.84) and 0.82 (0.81, 0.82, *I*^2^ = 98%) for random- and fixed-effects models, respectively (Figure 1A).

Because the primary analysis combined all EQ-5D data regardless of the version of the EQ-5D questionnaire, EQ-5D-3L or EQ-5D-5L, we performed a sensitivity analysis only using EQ-5D-5L,^17,21,23^ which had a pooled estimate (95% CI) of 0.79 (0.70, 0.87) and 0.76 (0.74, 0.77, I^2^ = 98%) in random- and fixed-effects models, respectively (Figure 1B). A sensitivity analysis for EQ-5D-3L was not feasible due to lack of data. Another sensitivity analysis was conducted, whereby we separated the combined EQ-5D data by study design (clinical trial or observational data). In the analysis where only clinical trial data were considered, the pooled estimate was 0.83 (0.82, 0.85) and 0.84 (0.83, 0.85, I^2^ = 77%) for random- and fixed-effects models, respectively (Figure 1C), and in the analysis where only observational data was considered, the pooled estimate was 0.76 (0.70, 0.83) and 0.74 (0.73, 0.75, I^2^ = 96%) for random- and fixed-effects models, respectively (Figure 1D). In both the primary and sensitivity analyses, the high I^2^ statistic indicates substantial heterogeneity, and therefore, the random-effects estimate should be considered.

#### EQ 5D second and later line

**Figure 2.**
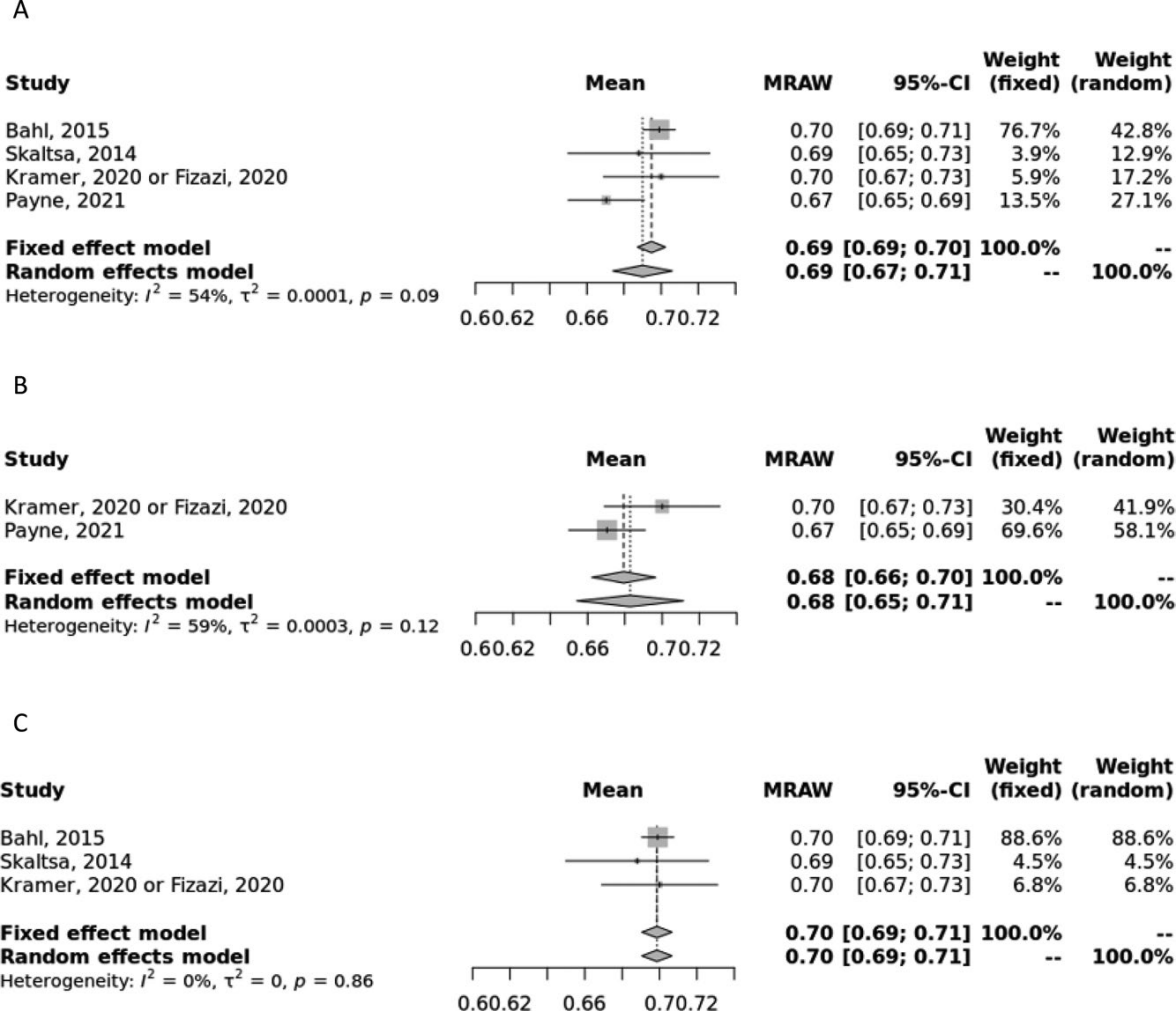
Meta-analysis of second line and later EQ-5D index scores. (A) Forest plot of combined second line and later EQ-5D-3L and EQ-5D-3L index scores in all primary studies. (B) Forest plot of only second line or later EQ-5D-5L index scores in all primary studies. (C) Forest plot of combined second line or later EQ-5D-3L and EQ-5D-5L index scores from clinical trials only. Abbreviation: MRAW, raw, untransformed mean.

In the 2L + setting, the pooled estimate for EQ-5D index utility values in the primary analysis was 0.69 (0.67, 0.71) and 0.69 (0.69, 0.70, I^2^ = 54%) for random- and fixed-effects models, respectively (Figure 2A).^23,28-30,34^

A sensitivity analysis only using EQ-5D-5L was conducted, and the pooled estimate was 0.68 (0.65, 0.71) and 0.68 (0.66, 0.70, I^2^ = 59%) for random- and fixed-effects models, respectively (Figure 2B),^23,29,30^; results were in line with the primary analysis. A sensitivity analysis for EQ-5D-3L was not feasible due to lack of data. In the sensitivity analysis where only clinical trial data were considered, the pooled estimate was 0.70 (0.69, 0.71) and 0.70 (0.69, 0.71, I^2^ = 0%) for random- and fixed-effects models, respectively (Figure 2C). A sensitivity analysis involving only observational data was not feasible due to lack of data. In the primary and EQ-5D-5L sensitivity analysis, the I^2^ statistic indicated moderate to substantial heterogeneity; therefore, the random-effects estimate should be considered. However, the I^2^ in the sensitivity analysis of clinical trial data indicated low heterogeneity, and therefore, the fixed-effects estimate can be considered.

#### EQ VAS first line

**Figure 3.**
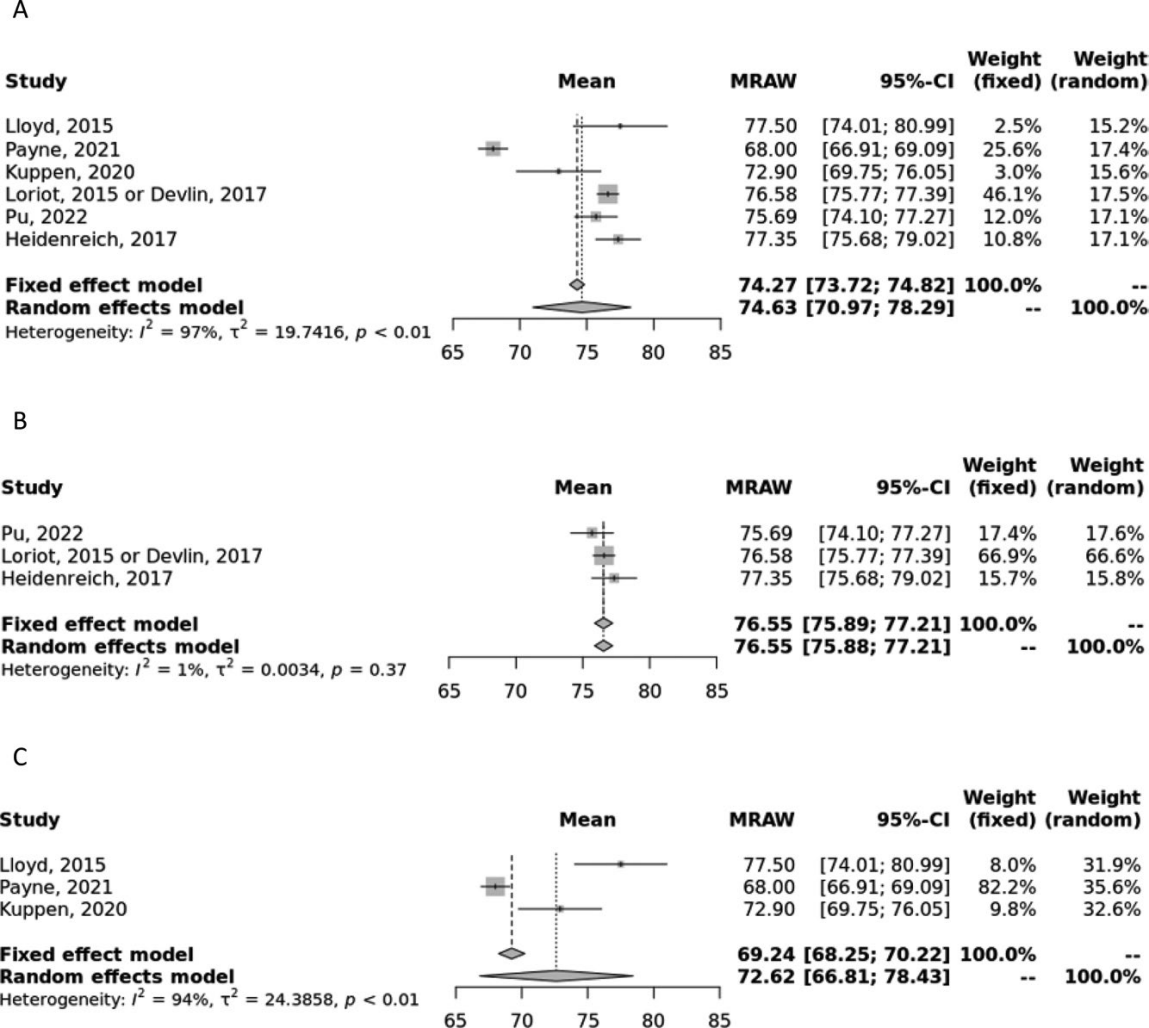
Meta-analysis of first-line EQ VAS scores. (A) Forest plot of first-line EQ VAS scores in all primary studies. (B) Forest plot of first-line EQ VAS scores from clinical trials only. (C) Forest plot of first-line EQ VAS scores from observational studies only. Abbreviation: MRAW, raw, untransformed mean.

The pooled estimates for 1L EQ VAS were 74.63 (70.97, 78.29) and 74.63 (73.72, 74.82, I^2^ = 97%) for random- and fixed-effects models, respectively (Figure 3A).^16-19,21,23,27^ The high I^2^ value indicates substantial heterogeneity, and therefore, the random-effects estimate should be considered.

In the analysis where only clinical trial data were considered, the pooled estimate was 76.55 (75.89, 77.21) for both random- and fixed-effects models, with an I^2^ of 0%, indicating low heterogeneity (Figure 3B). In the analysis where only observational data were considered, the pooled estimate was 72.62 (66.81, 78.43) and 69.24 (68.25, 70.22, I^2^ = 94%) for random- and fixed-effects models, respectively (Figure 3C). The high I^2^ indicates substantial heterogeneity, and therefore, the random-effects estimate should be considered.

#### EQ VAS second and later line

**Figure 4.**
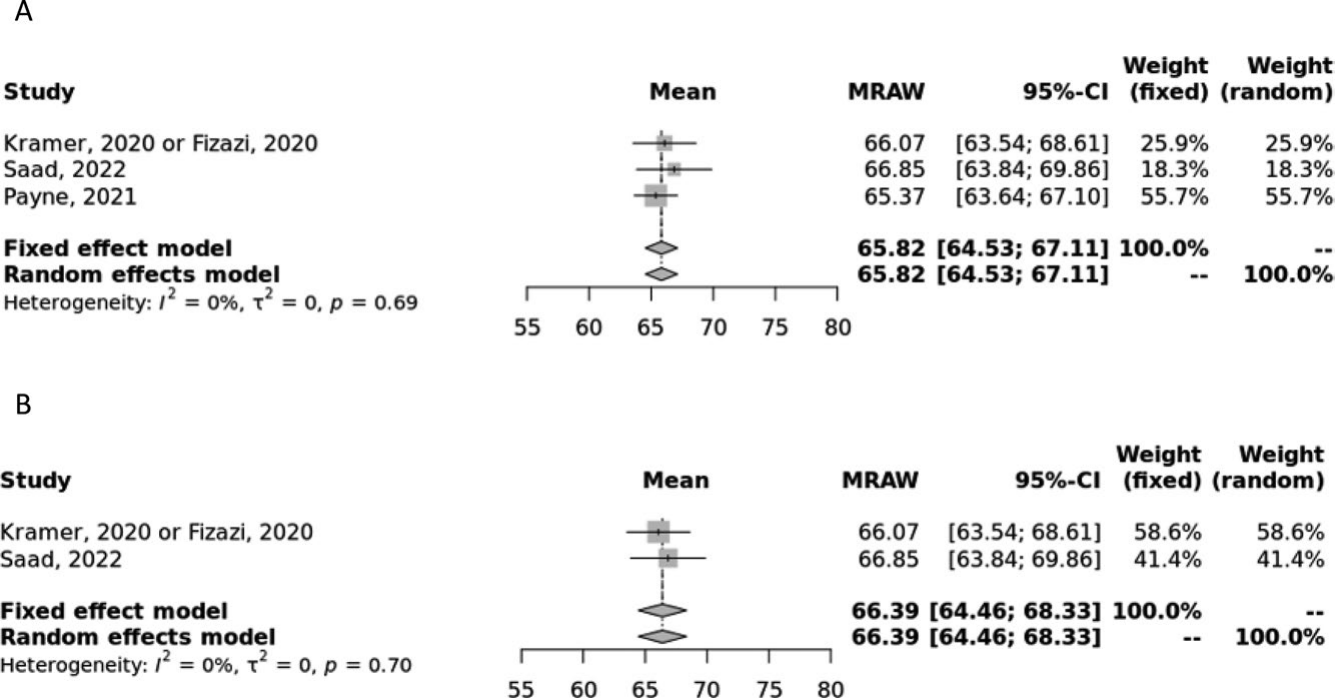
Meta-analysis of second line or later EQ VAS scores. (A) Forest plot of second line or later EQ VAS scores in all primary studies. (B) Forest plot of second line or later EQ VAS scores in clinical trials only. Abbreviations: CI, CI; MRAW, raw, untransformed mean.

In the 2L + setting, the pooled estimates for EQ VAS were 65.82 (64.53, 67.11, I^2^ = 0%) in both the random- and fixed-effects models, respectively (Figure 4A).^23,29–31^ The low I^2^ of 0% indicated low heterogeneity.

In a sensitivity analysis of clinical trial data only, the pooled estimate was 66.39 (64.46, 68.33) in both random- and fixed-effects models with an I^2^ of 0%, indicating low heterogeneity (Figure 4B). A sensitivity analysis for observational data only was not feasible due to lack of data.

## Discussion

This review focused on HSUV data across multiple lines of therapy and included both clinical trials and observational studies in patients with asymptomatic or mildly symptomatic mCRPC. We identified 46 unique studies reporting HSUV data, of which 12 were feasible for meta-analysis. The results of the narrative synthesis showed that EQ-5D values reported in 2L + were lower than 1L. This trend was further supported by the meta-analysis conducted on baseline EQ-5D index utility values and EQ VAS scores by line of therapy, where the pooled mean estimate of baseline EQ-5D index utility values and EQ VAS scores in 2L + was lower than the 1L setting. This is consistent with previous findings that HSUVs consistently decline as patients progress through worsening states of cancer (such as stage IV).^39^ This decline indicates a substantial impact on patient’s QoL, which underscores the clinical importance of early detection and intervention in the treatment of mCRPC. The differences in EQ-5D data presented in this SLR between lines of therapy can help guide clinicians, policymakers, and payers in optimizing individualized treatment strategies to improve patient QoL as they progress through lines of therapy. Current guidelines for the treatment of mCRPC generally recommend an individualized treatment approach, with abiraterone acetate with prednisone or enzalutamide often used as the preferred 1L therapy and cabazitaxel, radium-223, and PARP inhibitors often used in subsequent lines of therapy, with the American Society of Clinical Oncology/Cancer Care Ontario guidelines noting most these therapies (aside from cabazitaxel) as having demonstrated benefit to both survival and QoL.^40–42^ Additionally, sensitivity analyses including only baseline EQ-5D-5L index utility values or data from clinical or observational studies only were consistent with the primary analyses. In general, EQ-5D utility values or VAS scores derived from clinical trial data were slightly higher than those derived from observational data.

Recently published systematic reviews have focused on QoL in mCRPC^4,43^ and other PC-related disease stages (including metastatic hormone-naïve PC and non-mCRPC).^44^ Kretschmer et al conducted an SLR spanning approximately 8 years (from January 2011 through March 2019) of health-related QoL (HRQoL) outcomes in advanced PC (including mCRPC) and provided a qualitative synthesis of their findings. Nine studies in their review focused on patients with mCRPC and showed that first line use of enzalutamide, abiraterone acetate, or radium-223 was associated with positive HRQoL effects. Although this study provides a valuable summary of HRQoL recently, it focused on multiple clinical stages of advanced PC, was conducted in the first-line setting only, included only clinical trial data, and was qualitative only. Although our SLR identified the same studies as Kretschmer et al in asymptomatic/mildly symptomatic mCRPC, the objective of our study was to synthesize utility data to aid in informing inputs for future health economic decision models; therefore, all but Devlin et al^19^ were excluded from the present analysis. Ternov et al^4^ provided a narrative synthesis of their SLR and meta-analysis comparing short-term HRQoL (change in FACT-P from baseline to week 12) in patients receiving either enzalutamide or abiraterone acetate in first-line mCRPC. They reported pooled estimates of change from baseline of −1.3 (95% CI, −2.7 to 0.1) with enzalutamide and 4.7 (95% CI, −0.1 to 9.6) with abiraterone acetate in combination with prednisone. Although this study included data from both RCTs and observational studies, it had a narrower focus than our analysis—limiting the data to patients receiving enzalutamide or abiraterone acetate only and focusing on the FACT-P instrument for measuring QoL in first-line mCRPC, excluding measures of utility and later lines of therapy. Therefore, the results of this SLR and meta-analysis cannot be directly compared to our own. Of note, our review did capture the same studies as Ternov et al., and we too deemed it feasible to conduct a pooled analysis. Given the robust analysis presented by Ternov and colleagues, a re-analysis of the same evidence base would be redundant. McCool et al ^43^ conducted an SLR and network meta-analysis of various outcomes in mCRPC, including time to QoL deterioration, but not utility values. The scope of this study was to estimate the relative effects between treatments of interest and therefore cannot be directly compared to our present analysis. Although previous systematic literature reviews have studied QoL in mCRPC at the treatment level, the present SLR with meta-analysis addresses important gaps in the existing literature with a broader focus. Here we summarize QoL data and more specifically EQ-5D utility data in first and later lines of therapy in asymptomatic or mildly symptomatic patients with mCRPC by incorporating multiple sources, including data from economic evaluations and HTA submissions (and not just RCTs or observational studies) and focus strictly on utility values for meta-analysis rather than other measures of QoL to help inform future CUA models in support of HTA submissions. For example, the higher utility values observed in 1L therapy compared to 2L + in our analysis would benefit interventions that demonstrate higher progression-free survival, as this would increase the quality-adjusted life years and therefore the incremental cost-utility ratio in a CUA.

There are several strengths of the study presented, namely the robust search and meta-analysis methodology. First, compared to a traditional narrative review, this SLR provides a more comprehensive and thorough assessment of the existing research and offers a less biased perspective of the evidence, as studies are evaluated using a systematic approach and any reasons for exclusion are recorded during the full-text screening process. The subject of this meta-analysis, namely the focus on utility values and the inclusion of multiple lines of therapy and study types, offers a specific collection of data compared to the previously published SLRs on QoL in mCRPC described earlier. This study includes utility values from different health states, lines of therapy, and even utilities and incremental/decremental values associated with specific treatments, thus providing a valuable resource for researchers and health economists to accurately estimate utility values in CUAs and other analyses. Other strengths of this analysis include the de-duplication of utility sources from economic evaluations, meaning this synthesis represents only unique utility data, and that a homogenous population of only mCRPC patients is included. Finally, the methodology used in conducting the meta-analyses allows us to combine data in a unified pooled manner. Although other utility instruments are available for use, we chose to restrict our analysis to EQ-5D (and EQ VAS) to minimize heterogeneity and so results are most valid for CUAs. In addition, previous meta-analyses of utility values have cautioned against the pooling of utility values derived from different instruments.^45–47^ In most analyses, the random-effects model should be considered due to the inherent heterogeneity across the included studies; however, we provided both random- and fixed-effects estimates to demonstrate that despite the inherent heterogeneity, the estimates were quite similar (Figures 1-4).

Among the included studies, several data gaps do exist. One example includes the lack of reporting of the line of therapy or type of therapy given in many studies. In some cases, this data gap was to be expected. For example, economic evaluations use the same utility values for all treatments—differences in treatment are instead represented by the time spent in a certain health state. A small number of economic studies did provide a treatment-specific utility increment or decrement which was reported in Supplementary Table S14. Another gap in the data was the substantial heterogeneity in the reporting of utility values and VAS scores at study timepoints other than baseline. This data gap made it difficult to compare changes from baseline across studies in a meaningful way; however, differences between baseline 1L and 2L + utility values and VAS score ranges show a decline in QoL. This highlights the need for a more standardized reporting method for changes of utilities from baseline to allow for comparison. Finally, although the mCRPC treatment landscape is maturing with contemporary options, there was a dearth of studies included that investigated QoL with these contemporary treatments (such as PARP inhibitors) and whether these treatments are able to maintain QoL compared to their traditional counterparts. As more data for these contemporary treatments become available, it may warrant revisiting our analyses.

As with any study, our SLR and meta-analysis were not without limitations. First, caution must be used in interpreting the results of this SLR and meta-analysis to be solely attributed to the line of therapy or a particular health state of the patient. In this SLR, we were unable to capture the effect of treatment patterns/sequencing on QoL due to a lack of or inconsistent reporting of prior or subsequent therapy in the specific lines of therapy we analyzed. In general, more aggressive therapies are used in earlier lines of therapy rather than in later lines, which could contribute to reduced QoL in later lines of therapy. Therefore, a patient’s treatment journey could impact their present QoL as noted in a recent real-world study assessing mCRPC treatment patterns.^48^ Publication bias and selective reporting could also impact the interpretation of results in this SLR. For example, studies with positive or significant results are more likely to be published compared to null or negative results, and the absence of this evidence could lead to misleading results. To mitigate these risks, we included unpublished work from conference abstracts, government agencies, and trial registries and used quality assessment to detect bias in the included publications.

Second, many studies did not specify the EQ-5D instrument used (ie, EQ-5D-3L or EQ-5D-5L); therefore, utility values presented herein reflected either instrument, except for the sensitivity meta-analysis for which only the EQ-5D-5L scale was feasible. Despite many similarities between the EQ-5D-3L and EQ-5D-5L questionnaires, they differ in the scale that the questionnaire is answered—the EQ-5D-3L uses a 3-level scale (no problems, some problems, and extreme problems) vs the 5-level scale of the EQ-5D-5L (no problems, slight problems, moderate problems, severe problems, and extreme problems).^6,7^ Additionally, EQ-5D index utility values mapped from other QoL scales (such as FACT-P) may have used different mapping algorithms and may result in heterogeneity. Heterogeneity could also arise from the inclusion of studies from various geographical regions. Due to the paucity of data, it was not feasible to conduct country-specific analyses. The heterogeneity encountered by including any scale in the primary meta-analysis was partially addressed by conducting a random-effects meta-analysis and by performing a sensitivity analysis in which only EQ-5D-5L index values were included. A sensitivity analysis of only EQ-5D-3L values could not be performed due to lack of data. The results of the sensitivity analyses were consistent with the primary analyses. The meta-analysis was also limited in that no assumptions were made for statistical methodologies, missing data, or for outliers. However, because our analysis presents baseline utility data, few studies were missing data at this timepoint, and outliers were not considered due to the small number of studies included in the analysis.

Analysis of utility data from economic evaluations was often limited by the type of data available. Many economic evaluations did not report utility values for specific health states, were unclear regarding which health state the utility value represented, or had confidential/redacted utility value, and therefore, these studies were excluded from our results summary due to ambiguity. In addition, utility values reported in economic evaluations were generally not treatment-specific—they reported utilities by health state, and all treatments in that health state were given the same value.

Finally, although we analyzed baseline utility values in 1L and 2L + separately and noted lower values in the 2L + setting than 1L, these observations are based on snapshots from multiple studies with different patient populations and different therapeutic strategies. Our meta-analysis took a treatment-agnostic approach as treatment-specific analyses were not feasible due to the paucity of data available for each of the included treatment modalities. Ideally, studies should look at patients transitioning through health states or lines of therapy to accurately compare utility data in these health states and lines of therapy. Only one included study investigated the utility values of a single treatment used across different lines of therapy, and it had results consistent with those in this SLR.^23^

Future studies investigating utility values in mCRPC could focus on developing more standardized minimal clinically important differences for various utility instruments and protocols for the standardized reporting of utility values and QoL instruments to allow for stronger comparisons to be developed. With more standardized reporting of utility values and additional published data available for treatments in mCRPC, a future SLR could attempt to compare EQ-5D utility values between treatment options, especially since the management of mCRPC has evolved in the past decade due to substantial advances in understanding the genomic landscape and biology underpinning this form of PC. Additionally, intervention-specific longitudinal cohort studies investigating utility and QoL data trends over the course of a patient’s treatment journey could allow for a more accurate comparison of utility values in a certain health state or line of therapy and address the heterogeneity in utility data found in our analysis.

## Conclusion

In conclusion, this systematic review with meta-analysis comprehensively identified and synthesized utility values in the form of EQ-5D and EQ VAS in patients with asymptomatic or mildly symptomatic mCRPC by line of therapy and incorporated both clinical trial data and observational evidence. Overall, notably lower utility values in the 2L + mCRPC setting compared with the 1L setting were found, highlighting the need for early detection and earlier introduction of effective treatments in the disease pathway of mCRPC. The results of these analyses can act as a valuable resource for those developing CUAs and other analyses related to QoL to support drug reimbursement in mCRPC and highlight the need for more standardized QoL reporting.

## Supplementary Material

oyae321_suppl_Supplementary_Material

## Data Availability

All data on which the conclusion is based can be accessed in the Supplementary Materials or manuscript.
