# Supplementary material for: Systematic literature review and meta-analysis of health state utility values in metastatic castration-resistant prostate cancer
Source: Oncologist. 2024 Nov 26;30(7):oyae321. doi: 10.1093/oncolo/oyae321 (PMC12311294; doi:10.1093/oncolo/oyae321)
Supplement: oyae321_suppl_Supplementary_Material [file oyae321_suppl_supplementary_material.docx]

# Supplementary Materials

## Supplementary Materials A: Material and Methods

## Systematic Literature Search

The MEDLINE® search strategy was peer-reviewed independently by another senior medical information specialist before execution using the Peer Review of Electronic Search Strategies (PRESS) checklist.^1^ ^2^ Language was restricted to English-only articles. Animal-only and opinion pieces were removed from the results. The original search was not limited to specific dates, the databases were searched from inception to present.

The bibliographies of relevant published reviews were searched to validate findings from the database searches. The initial search was conducted on 2021 August 19 and updated on 2022 October 3 to include information that had been published since the initial search. The search strategies are detailed in **Supplementary Table S2 and Table S3 in Supplemental Materials.**

**Study Selection and Data Extraction**

All studies retrieved from the database searches were imported into EndNote Version 20.1 and 20.4 (Clarivate, EndNote, Chandler, AZ, USA) for the deduplication process and subsequently imported into DistillerSR Version 2.35 and 2.41 (DistillerSR Inc. 2021, Ottawa, Canada) for further deduplication and screening. Screening was performed according to the PICOS criteria in two levels by two independent reviewers – first at the title and abstract level and then in full-text to determine formal inclusion into the review and data extraction. Eligibility was determined by consensus of the two reviewers; a third reviewer was consulted to resolve any discrepancies as required.

**Quality Assessment**

^34^Quality assessments were only completed for full-text publications, not conference abstracts, as they did not have sufficient methodological data to assess the study. Study quality assessment was conducted by a single reviewer and a second reviewer validated consistency and integrity of the assessments.

## Meta-Analysis

Some studies contributed multiple treatment groups to the meta-analyses. Though it was determined that all treatment groups from the same study were independent, there are subtle complications in simply including all treatment arms as if they were different studies with regards to study-to-study variation. To avoid double-counting contributions to between-study heterogeneity in random-effects meta-analyses, a fixed-effect meta-analysis was performed separately for each study contributing multiple treatment groups. The resulting pooled treatment effects and standard errors were then used to conduct a random-effects meta-analysis across studies, as suggested by Cochrane.^5^ Results presented for the meta-analysis summary is based on heterogeneity according to the I^2^ statistic – fixed-effect estimates were presented for analyses with low heterogeneity and random-effects analyses were presented for studies with high heterogeneity. Both fixed-effect and random-effects estimates are available in the forest plots and in-text results. Heterogeneity was classified as low if I^2^ < 40%, moderate if 30% < I^2^ < 60%, substantial if 50% < I^2^ < 90% and high if I^2^ > 75%.^6^

## Supplementary Materials A: Results

## Summary from Primary Studies

### Study Characteristics

The 1L primary studies spanned several geographic regions including North America and the European Union (EU) (n=3 studies)^7-10^, EU only (n=3)^11-13^, UK only (n=1 study)^14^, Italy only (n=1 study)^15, 16^, Netherlands only (n=1 study)^17^, and in East Asia (n=1 study).^18^ The population sizes ranged from 20 participants in cross-sectional studies to >1,000 in clinical trials. In the 1L setting, enzalutamide was the most common intervention, with six studies reporting utilities for this treatment.^7-10, 12, 13, 18^ Two studies reported utility values for patients receiving abiraterone acetate (with or without prednisone),^13, 15, 16^ and one study for docetaxel.^14^ Two studies did not specify which intervention was used (**Supplementary Table S8**).^11, 17^

The 2L+ studies were conducted across various geographical settings. Half of the 2L+ studies took place globally,^19-23^ with the EU,^12^ UK,^24^ and Australia,^25^ each represented by a single included study. The number of participants ranged from ~100 to >500. The treatments evaluated in the 2L+ settings included cabazitaxel (n=3 studies), ^19, 20, 24, 25^ enzalutamide (n=2 studies),^12, 19, 20^ abiraterone acetate (with or without prednisone) (n=2 studies),^19-21^ and poly ADP ribose polymerase (PARP) inhibitors (n=2 studies).^21, 22^ One study investigated an unspecified treatment as part of their mapping analysis (**Supplementary Table S8**).^23^

### EQ-5D and VAS Data from Primary Studies by Treatment

In the 1L setting, baseline mean EQ-5D index utility values and EQ VAS score were reported in six studies evaluating enzalutamide,^7-10, 12, 13, 18^ two studies evaluating abiraterone acetate (with or without prednisone),^13, 15, 16^ and one study evaluating docetaxel.^14^ In the 2L+ setting, baseline mean EQ-5D index utility values and EQ VAS scores were reported in two studies evaluating cabazitaxel,^19, 20, 24^ two studies evaluating abiraterone acetate (with or without prednisone),^19-21^ two studies evaluating enzalutamide,^12, 19, 20^ and one study evaluating olaparib (**Supplementary Table S11**).^21^ The EQ-5D index utility values between treatments in 1L were generally less consistent than in the 2L+ setting; however, EQ VAS scores were more consistent across specific treatments used in 1L or 2L+ (**Supplementary Table S11**). Where data were available for a specific treatment in both 1L and 2L+, a lower in EQ-5D index utility value and EQ VAS score was observed in 2L+ compared with 1L (**Supplementary Table S11**).

## Summary from Economic Evaluations

### Study Characteristics

Most of the economic evaluations were performed from either a US or United Kingdom perspective^26-50^; however, studies from Canada^51-53^, Japan^54^, China^55^, and the Netherlands^56^ were also represented (**Supplementary Table S9-S10**).

These studies reported EQ-5D index utility values by health state and by line of therapy. The interventions reflective of the 1L setting were abiraterone acetate (with or without prednisone), enzalutamide, docetaxel, and sipuleucel T. Treatments reflective of the 2L+ setting included abiraterone acetate (with or without prednisone), enzalutamide, cabazitaxel, and olaparib. Health states across these studies were categorized into stable disease (SD) or progression-free survival (PFS), progressed disease (PD), palliative/end-of-life care, and death (**Supplementary Table S9-S10**).

### EQ-5D Data from Economic Studies by Health State

In the 1L setting, the range of HSUVs reported for SD/PFS ^26, 35-38, 47, 51, 54, 56^ were 0.63 – 0.844, which were higher than those reported for PD – 0.65 – 0.715 ^26, 37, 38, 56^, indicating a decline in health as a patient moves to a more progressive disease stage (**Supplementary Table S12**).

A similar trend was observed in the assessment of the 2L+ setting – the range of HSUVs reported for SD/PFS ^27-30, 34, 37, 38, 40, 41, 44, 45, 47, 48, 51, 54, 55^ were higher than those for PD (0.617 – 0.83 versus 0.37 – 0.65, respectively) (**Supplementary Table S12**).^27-30, 34, 40, 41, 44, 45, 48, 49, 55^

Utility values used in economic evaluations can also vary by the geographical region they are representing. This is highlighted in a Dutch study, where it was noted that the Dutch population has substantially higher QoL than other populations, and therefore, higher utility values associated with certain health states.^56^ Due to the paucity of data, it was not feasible to conduct country-specific analyses. However, when utility values from this Dutch economic evaluation were removed, the values encompassed by the range were lower, especially in the progressed disease health state (**Supplementary Table S13**).

## Supplementary Materials A: References

1. McGowan J, Sampson M, Salzwedel DM, Cogo E, Foerster V, Lefebvre C. PRESS Peer Review of Electronic Search Strategies: 2015 Guideline Statement. *J Clin Epidemiol* **75**, 40-6 (2016)

2. Arber M, Garcia S, Veale T, Edwards M, Shaw A, Glanville JM. PERFORMANCE OF OVID MEDLINE SEARCH FILTERS TO IDENTIFY HEALTH STATE UTILITY STUDIES. *Int J Technol Assess Health Care* **33**(4), 472-80 (2017)

3. Papaioannou D, Brazier J, Paisley S. NICE Decision Support Unit Technical Support Documents. NICE DSU Technical Support Document 9: The Identification, Review and Synthesis of Health State Utility Values from the Literature. (National Institute for Health and Care Excellence (NICE) Copyright © 2010 National Institute for Health and Clinical Excellence, unless otherwise stated. All rights reserved., London, 2010).

4. Drummond MF, Jefferson TO. Guidelines for authors and peer reviewers of economic submissions to the BMJ. The BMJ Economic Evaluation Working Party. *Bmj* **313**(7052), 275-83 (1996)

5. Cochrane Statistical Methods Group. Chapter 23: Including variants on randomized trials. In: Julian PT Higgins SE, Tianjing Li, editor. Cochrane Handbook for Systematic Reviews of Interventions version 63. (Cochrane, <https://training.cochrane.org/handbook/current/chapter-23>, 2022).

6. Cochrane Statistical Methods Group. Chapter 10: Analysing data and undertaking meta-analyses. In: Deeks JJ HJ, Altman DG, editor. Cochrane Handbook for Systematic Reviews of Interventions version 63. (Cochrane, <https://training.cochrane.org/handbook/current/chapter-10>, 2022).

7. Heidenreich A, Chowdhury S, Klotz L, Siemens DR, Villers A, Ivanescu C, et al. Impact of Enzalutamide Compared with Bicalutamide on Quality of Life in Men with Metastatic Castration-resistant Prostate Cancer: Additional Analyses from the TERRAIN Randomised Clinical Trial. *Eur Urol* **71**(4), 534-42 (2017)

8. Loriot Y, Miller K, Sternberg CN, Fizazi K, De Bono JS, Chowdhury S, et al. Effect of enzalutamide on health-related quality of life, pain, and skeletal-related events in asymptomatic and minimally symptomatic, chemotherapy-naive patients with metastatic castration-resistant prostate cancer (PREVAIL): results from a randomised, phase 3 trial. *Lancet Oncol* **16**(5), 509-21 (2015)

9. Devlin N, Herdman M, Pavesi M, Phung D, Naidoo S, Beer TM, et al. Health-related quality of life effects of enzalutamide in patients with metastatic castration-resistant prostate cancer: an in-depth post hoc analysis of EQ-5D data from the PREVAIL trial. *Health Qual Life Outcomes* **15**(1), 130 (2017)

10. Saad F, Ivanescu C, Phung D, Loriot Y, Abhyankar S, Beer TM, et al. Skeletal-related events significantly impact health-related quality of life in metastatic castration-resistant prostate cancer: data from PREVAIL and AFFIRM trials. *Prostate Cancer Prostatic Dis* **20**(1), 110-6 (2017)

11. Diels J, Hamberg P, Ford D, Price PW, Spencer M, Dass RN. Mapping FACT-P to EQ-5D in a large cross-sectional study of metastatic castration-resistant prostate cancer patients. *Qual Life Res* **24**(3), 591-8 (2015)

12. Payne H, Robinson A, Rappe B, Hilman S, De Giorgi U, Joniau S, et al. A European, prospective, observational study of enzalutamide in patients with metastatic castration-resistant prostate cancer: PREMISE. *Int J Cancer* **150**(5), 837-46 (2022)

13. Dearden L, Shalet N, Artenie C, Mills A, Jackson C, Grant L, et al. Fatigue, treatment satisfaction and health-related quality of life among patients receiving novel drugs suppressing androgen signalling for the treatment of metastatic castrate-resistant prostate cancer. *Eur J Cancer Care (Engl)* **28**(1), e12949 (2019)

14. Lloyd AJ, Kerr C, Penton J, Knerer G. Health-Related Quality of Life and Health Utilities in Metastatic Castrate-Resistant Prostate Cancer: A Survey Capturing Experiences from a Diverse Sample of UK Patients. *Value Health* **18**(8), 1152-7 (2015)

15. Procopio G, Chiuri VE, Giordano M, Alitto AR, Maisano R, Bordonaro R, et al. Real-world experience of abiraterone acetate plus prednisone in chemotherapy-naive patients with metastatic castration-resistant prostate cancer: long-term results of the prospective ABItude study. *ESMO Open* **7**(2), 100431 (2022)

16. Procopio G, Chiuri VE, Giordano M, Mantini G, Maisano R, Bordonaro R, et al. Effectiveness of abiraterone acetate plus prednisone in chemotherapy-naïve patients with metastatic castration-resistant prostate cancer in a large prospective real-world cohort: the ABItude study. *Ther Adv Med Oncol* **12**, 1758835920968725 (2020)

17. Kuppen MCP, Westgeest HM, van den Eertwegh AJM, Coenen J, van Moorselaar RJA, van den Berg P, et al. Health-related Quality of Life and Pain in a Real-world Castration-resistant Prostate Cancer Population: Results From the PRO-CAPRI Study in the Netherlands. *Clin Genitourin Cancer* **18**(3), e233-e53 (2020)

18. Pu YS, Ahn H, Han W, Huang SP, Wu HC, Ma L, et al. Enzalutamide in Chemotherapy-Naïve Metastatic Castration-Resistant Prostate Cancer: An Asian Multiregional, Randomized Study. *Adv Ther* **39**(6), 2641-56 (2022)

19. Kramer* G, Sternberg CN, Fizazi K, Tombal B, Eymard J-C, Bono JD, et al. PD16-08 EFFECT OF CABAZITAXEL VS ABIRATERONE OR ENZALUTAMIDE ON PATIENT-REPORTED OUTCOMES IN METASTATIC CASTRATION-RESISTANT PROSTATE CANCER: A PRE-PLANNED EQ-5D-5L ANALYSIS OF THE CARD STUDY. *Journal of Urology* **203**(Supplement 4), e366-e (2020)

20. Fizazi K, Kramer G, Eymard JC, Sternberg CN, de Bono J, Castellano D, et al. Quality of life in patients with metastatic prostate cancer following treatment with cabazitaxel versus abiraterone or enzalutamide (CARD): an analysis of a randomised, multicentre, open-label, phase 4 study. *Lancet Oncol* **21**(11), 1513-25 (2020)

21. Saad F, Thiery-Vuillemin A, Wiechno P, Alekseev B, Sala N, Jones R, et al. Patient-reported outcomes with olaparib plus abiraterone versus placebo plus abiraterone for metastatic castration-resistant prostate cancer: a randomised, double-blind, phase 2 trial. *Lancet Oncol* **23**(10), 1297-307 (2022)

22. Saad F, de Bono J, Barthélémy P, Dorff T, Mehra N, Scagliotti G, et al. Patient-reported Outcomes in Men with Metastatic Castration-resistant Prostate Cancer Harboring DNA Damage Response Alterations Treated with Talazoparib: Results from TALAPRO-1. *Eur Urol* **83**(4), 352-60 (2023)

23. Skaltsa K, Longworth L, Ivanescu C, Phung D, Holmstrom S. Mapping the FACT-P to the preference-based EQ-5D questionnaire in metastatic castration-resistant prostate cancer. *Value Health* **17**(2), 238-44 (2014)

24. Bahl A, Masson S, Malik Z, Birtle AJ, Sundar S, Jones RJ, et al. Final quality of life and safety data for patients with metastatic castration-resistant prostate cancer treated with cabazitaxel in the UK Early Access Programme (EAP) (NCT01254279). *BJU Int* **116**(6), 880-7 (2015)

25. Parente P, Ng S, Parnis F, Guminski A, Gurney H. Cabazitaxel in patients with metastatic castration-resistant prostate cancer: safety and quality of life data from the Australian early access program. *Asia Pac J Clin Oncol* **13**(6), 391-9 (2017)

26. Gong CL, Hay JW. Cost-effectiveness analysis of abiraterone and sipuleucel-T in asymptomatic metastatic castration-resistant prostate cancer. *J Natl Compr Canc Netw* **12**(10), 1417-25 (2014)

27. Zhang PF, Xie D, Li Q. Cost-effectiveness analysis of cabazitaxel for metastatic castration resistant prostate cancer after docetaxel and androgen-signaling-targeted inhibitor resistance. *BMC Cancer* **21**(1), 35 (2021)

28. Ko G, Hansen R. PCN178 Cost-Effectiveness Analysis of Olaparib and Novel Hormonal Therapies in Metastatic Castration Resistant Prostate Cancer. *Value in Health* **24**, S52 (2021)

29. Su D, Wu B, Shi L. Cost-Effectiveness of Genomic Test-Directed Olaparib for Metastatic Castration-Resistant Prostate Cancer. *Front Pharmacol* **11**, 610601 (2020)

30. Barqawi YK, Borrego ME, Roberts MH, Abraham I. Cost-effectiveness model of abiraterone plus prednisone, cabazitaxel plus prednisone and enzalutamide for visceral metastatic castration resistant prostate cancer therapy after docetaxel therapy resistance. *J Med Econ* **22**(11), 1202-9 (2019)

31. Wilson L, Tang J, Zhong L, Balani G, Gipson G, Xiang P, et al. New therapeutic options in metastatic castration-resistant prostate cancer: Can cost-effectiveness analysis help in treatment decisions? *J Oncol Pharm Pract* **20**(6), 417-25 (2014)

32. Holko P, Kawalec P. Economic evaluation of sipuleucel-T immunotherapy in castration-resistant prostate cancer. *Expert Rev Anticancer Ther* **14**(1), 63-73 (2014)

33. Zhong L, Pon V, Srinivas S, Nguyen N, Frear M, Kwon S, et al. Therapeutic options in docetaxel-refractory metastatic castration-resistant prostate cancer: a cost-effectiveness analysis. *PLoS One* **8**(5), e64275 (2013)

34. Li Y, Lin S, Zhong L, Luo S, Huang X, Huang X, et al. Is olaparib cost effective in metastatic castration-resistant prostate cancer patients with at least one favorable gene mutation in BRCA1, BRCA2 or ATM? *Pharmacogenomics* **22**(13), 809-19 (2021)

35. NICE. Final Appraisal Determination - Abiraterone for treating metastatic hormone-relapsed prostate cancer before chemotherapy is indicated. 2016. p. 1-54.

36. NICE. Technology Appraisal Guidance - Abiraterone for treating metastatic hormone-relapsed prostate cancer before chemotherapy is indicated. 2016. p. 1-52.

37. NICE. Final Appraisal Determination - Enzalutamide for treating metastatic hormone-relapsed prostate cancer before chemotherapy is indicated. 2015. p. 1-55.

38. NICE. Technology Appraisal Guidance - Enzalutamide for treating metastatic hormone-relapsed prostate cancer before chemotherapy is indicated. 2016. p. 1-48.

39. NICE. Single Technology Appraisal - Olaparib for previously treated hormone-relapsed metastatic prostate cancer (Committee Papers). 2020. p. 1-782.

40. NICE. Final Appraisal Determination - Abiraterone for castration-resistant metastatic prostate cancer previously treated with a docetaxel-containing regimen. 2012. p. 1-55.

41. NICE. Technology Appraisal Guidance - Abiraterone for castration-resistant metastatic prostate cancer previously treated with a docetaxel-containing regimen. 2012. p. 1-54.

42. NICE. Final Appraisal Determination - Enzalutamide for metastatic hormone-relapsed prostate cancer previously treated with a docetaxel-containing regimen. 2014. p. 1-65.

43. NICE. Technology Appraisal Guidance - Enzalutamide for metastatic hormone-relapsed prostate cancer preivously treated with a docetaxel-containing regimen. 2014. p. 1-62.

44. NICE. Final Appraisal Determination - Cabazitaxel for hormone-relapsed metastatic prostate cancer treated with docetaxel. 2016. p. 1-54.

45. NICE. Technology Appraisal Guidance - Cabazitaxel for hormone-relapsed metastatic prostate cancer treated with docetaxel. 2016. p. 1-48.

46. NICE. Technolgoy Appraisal Guidance - Olaparib for previously treated BRCA mutation-positive hormone-relapsed metastatic prostate cancer. 2022. p. 1-37.

47. SMC. Independent Review Panel - Abiraterone acetate 250mg tablets. 2015. p. 1-13.

48. SMC. 2nd Re-Submission - Cabazitaxel 60mg concentrate and solvent for solution for infusion. 2016. p. 1-11.

49. SMC. Enzalutamide 40mg soft capsules. 2013. p. 1-10.

50. Collins R, Fenwick E, Trowman R, Perard R, Norman G, Light K, et al. A systematic review and economic model of the clinical effectiveness and cost-effectiveness of docetaxel in combination with prednisone or prednisolone for the treatment of hormone-refractory metastatic prostate cancer. *Health Technol Assess* **11**(2), iii-iv, xv-xviii, 1-179 (2007)

51. Hill J, Paulden M, McCabe C, North SA, Venner P, Usmani N. Cost-effectiveness analysis of metformin with enzalutamide in the metastatic castrate-resistant prostate cancer setting. *Can J Urol* **26**(6), 10045-53 (2019)

52. Sanyal C, Aprikian AG, Cury FL, Chevalier S, Dragomir A. Management of localized and advanced prostate cancer in Canada: A lifetime cost and quality-adjusted life-year analysis. *Cancer* **122**(7), 1085-96 (2016)

53. Sanyal C, Aprikian A, Cury F, Chevalier S, Dragomir A. Clinical management and burden of prostate cancer: a Markov Monte Carlo model. *PLoS One* **9**(12), e113432 (2014)

54. Okumura H, Inoue S, Naidoo S, Holmstrom S, Akaza H. Cost-effectiveness analysis of enzalutamide for patients with chemotherapy-naïve metastatic castration-resistant prostate cancer in Japan. *Jpn J Clin Oncol* **51**(8), 1319-29 (2021)

55. Xu C, Cai J, Zhuang J, Zheng B, Chen L, Sun H, et al. Cost-effectiveness of olaparib, a PARP inhibitor, for patients with metastatic castration-resistant prostate cancer in China and United States. *Ann Transl Med* **10**(15), 830 (2022)

56. Ten Ham RMT, van Nuland M, Vreman RA, de Graaf LG, Rosing H, Bergman AM, et al. Cost-Effectiveness Assessment of Monitoring Abiraterone Levels in Metastatic Castration-Resistant Prostate Cancer Patients. *Value Health* **24**(1), 121-8 (2021)

## Supplementary Materials A: Tables

Table S1: PICOS Criteria

|  | Inclusion Criteria | Exclusion Criteria |
| --- | --- | --- |
| *Population* | Study populations or subgroups of patients (humans only; men) with:   - Age ≥18 years - Histologically or cytologically confirmed adenocarcinoma of the prostate - Undergone surgical or medical castration - Metastatic disease^A^ - Castration-resistant/Hormone-resistant/Hormone-refractory/Androgen-independent prostate cancer - Asymptomatic or mildly symptomatic mCRPC^A,B,C^ - Treatment naïve in the mCRPC setting | Study populations or subgroups:   - Non-human - Age <18 years - No surgical or medical castration - Non-metastatic disease - Non-CRPC - Hormone-sensitive disease - Any previous systemic cancer treatment for mCRPC disease state^H^ |
| *Interventions* | Any treatments available or under investigation for mCRPC provided as a single agent or a combination treatment^D,E^ | Those not listed in the inclusion criteria |
| *Comparators* | Any treatments available or under investigation for mCRPC provided as a single-agent or combination treatment^D,E^, or best supportive care, placebo, or watchful waiting | Those not listed in the inclusion criteria |
| *Outcomes* | - Generic preference-based HSUVs from the following instruments: EQ-5D-3L, EQ-5D-5L, SF-6D, HUI3, QWB index, 15D, AQoL - Generic measures from the following instruments: SF-36 - Disease-specific measures from the following instruments: EORTC QLQ-C30, EORTC QLQ-PR25, FACT-G, FACT-P - Values generated by measures which might be mapped on to the EQ-5D that are not already included above - Mapping algorithms of measurement instruments used in mCRPC to derive HSUVs | Those not listed in the inclusion criteria |
| *Study Design* | - RCTs irrespective of blinding - Non-randomized clinical trials - Observational studies (eg. Registry studies, prospective and retrospective cohort studies, cross-sectional studies) - Any HSUV elicitation studies (eg. TTO, SG, VAS) - Economic evaluations: cost-effectiveness studies and cost-utility studies - Conference abstracts - Assessment from HTA agencies when a reviewer’s full reports are available | - Pre-clinical studies, pharmacokinetic studies - Case study, series, or reports - Expert opinion articles - Letters, editorials - Narrative (non-systematic) reviews - Pilot studies - Protocols - SLRs^F^ |
| *Language* | Articles in English^G^ | All non-English articles |
| *Dates* | Database search: inception - present  Conference abstracts: 2019 to present | Database search: none  Conference abstracts: prior to 2019 |

^A^ Search strategy will not be limited to metastatic disease, by line of treatment, or severity of disease; however, studies reporting solely non-metastatic patients will be excluded during the screening phase

^B^ The primary population of interest will be asymptomatic or mildly symptomatic patients who are treatment naïve or patients with later line mCRPC

^C^ The definition of symptomatic disease will not be restricted, and definitions based on pain and/or other symptoms will be included. Differing definitions will be evaluated at the feasibility assessment stage

^D^ Androgen deprivation therapy is not exclusionary

^E^ Treatments under investigation for mCRPC include, but are not limited to poly (ADP-ribose) polymerase (PARP) inhibitors, chemotherapy agents, immunotherapies, novel hormone therapies, radiotherapy, ipataserib, etc.

^F^ Relevant SLRs will not be included in the final included studies list; however, their bibliographies will be reviewed for any additional relevant studies

^G^ Search strategy will not be limited by language, however, non-English articles will be excluded during the screening phase

^H^ Androgen deprivation therapy is not exclusionary.

Abbreviations: AQoL = Assessment of Quality of Life; EORTC QLQ-PR25 = European Organisation for Research and Treatment of Cancer Quality of Life Questionnaire - Prostate Cancer Module; EQ-5D = EuroQuol 5-Dimension; EQ-5D-3L = EuroQuol 5-Dimension 3-Level; EQ-5D-5L = EuroQuol 5-Dimension 5-Level; FACT-G = Functional Assessment of Cancer Therapy-General; FACT-P = Functional Assessment of Cancer Therapy-Prostate; HSUVs = health-state utility values; HUI3 = health utilities index mark 3; QWB = Quality of Well-Being; SF-36 = 36-Item Short Form Health Survey; RCTs = randomized controlled trials; SF-6D = Short-Form Six-Dimensions; SG = standard gamble; TTO = time trade-off; VAS = visual analogue scale.

Table S2: Search Strategy (August 19, 2021)

| **#** | **Searches** | **Results** |
| --- | --- | --- |
| 1 | Prostatic Neoplasms/ or (((prostate or prostatic) adj3 (adenocarcinoma$ or adeno-carcinoma$ or cancer$ or carcinoma$ or malignan$ or neoplas$ or tumor? or tumour?)) or PC or PCa).ti,ab,kf. | 659993 |
| 2 | Orchiectomy/ and (insensitiv$ or refractor$ or resistan$).ti,ab,kf. | 2327 |
| 3 | (((androgen? or castrat$ or hormon$) adj2 (independen$ or insensitiv$ or refractor$ or resistan$)) or ((orchectom$ or orcheotom$ or orchidectom$ or orchiectom$ or testectom$ or (removal adj3 (testicle? or test#s))) and (insensitiv$ or refractor$ or resistan$))).ti,ab,kf. | 64006 |
| 4 | 1 and (2 or 3) | 47724 |
| 5 | Prostatic Neoplasms, Castration-Resistant/ or (MCRPC or CRPC or MCRPCa or CRPCa or (((prostate or prostatic) adj3 (adenocarcinoma$ or adeno-carcinoma$ or cancer$ or carcinoma$ or malignan$ or neoplas$ or tumor? or tumour?)) and ((androgen? or castrat$ or hormon$) adj2 (independen$ or insensitiv$ or refractor$ or resistan$)))).ti,ab,kf. | 50611 |
| 6 | 4 or 5 | 50888 |
| 7 | exp Animals/ not Humans/ | 16122182 |
| 8 | 6 not 7 [ANIMAL-ONLY REMOVED] | 38903 |
| 9 | (comment or editorial or news or newspaper article).pt. | 2230772 |
| 10 | (letter not (letter and randomized controlled trial)).pt. | 2330688 |
| 11 | 8 not (9 or 10) [OPINION PIECES REMOVED] | 37487 |
| 12 | Quality-Adjusted Life Years/ | 44522 |
| 13 | (quality adjusted or adjusted life year$).ti,ab,kf. | 52196 |
| 14 | (qaly$ or qald$ or qale$ or qtime$).ti,ab,kf. | 38904 |
| 15 | (illness state$1 or health state$1).ti,ab,kf. | 21027 |
| 16 | (hui or hui1 or hui2 or hui3).ti,ab,kf. | 4576 |
| 17 | (multiattribute$ or multi attribute$).ti,ab,kf. | 2335 |
| 18 | (utility adj3 (score$1 or valu$ or health$ or cost$ or measur$ or disease$ or mean or gain or gains or index$)).ti,ab,kf. | 48148 |
| 19 | utilities.ti,ab,kf. | 21987 |
| 20 | (eq-5d or eq5d or eq-5 or eq5 or euro qual or euroqual or euro qual5d or euroqual5d or euro qol or euroqol or euro qol5d or euroqol5d or euro quol or euroquol or euro quol5d or euroquol5d or eur qol or eurqol or eur qol5d or eur qol5d or eur?qul or eur?qul5d or euro$ quality of life or European qol).ti,ab,kf. | 49129 |
| 21 | (euro$ adj3 (5 d or 5d or 5 dimension$ or 5dimension$ or 5 domain$ or 5domain$)).ti,ab,kf. | 15511 |
| 22 | (sf36$ or sf 36$ or sf thirtysix or sf thirty six).ti,ab,kf. | 77049 |
| 23 | (time trade off$1 or time tradeoff$1 or tto or timetradeoff$1).ti,ab,kf. | 5362 |
| 24 | quality of life/ and ((quality of life or qol) adj (score$1 or measure$1)).ti,ab,kf. | 43985 |
| 25 | quality of life/ and ec.fs. | 59831 |
| 26 | quality of life/ and (health adj3 status).ti,ab,kf. | 28236 |
| 27 | (quality of life or qol).ti,ab,kf. and Cost-Benefit Analysis/ | 21925 |
| 28 | or/12-27 [Arber 2017 Utilities filter - balanced] | 348193 |
| 29 | ("European Quality of Life questionnaire 5D" or "disability adjusted life" or "sickness impact profile" or daly$ or (short form 36 or shortform 36 or shortform thirtysix or shortform thirty six or short form thirtysix or short form thirty six) or (sf6 or sf 6 or short form 6 or shortform 6 or sf six or sfsix or shortform six or short form six) or (sf12 or sf 12 or short form 12 or shortform 12 or sf twelve or sftwelve or shortform twelve or short form twelve) or (sf6D or sf 6D or short form 6D or shortform 6D or sf six D or sfsixD or shortform six D or short form six D) or (sf20 or sf 20 or short form 20 or shortform 20 or sf twenty or sftwenty or shortform twenty or short form twenty) or (hql or hqol or h qol or hrqol or hr qol) or (hye or hyes) or health$ year$ equivalent$ or disutili$ or rosser or (quality adj2 wellbeing) or qwb or standard gamble$).ti,ab. or (SG and gamble).ab. [ADDITIONAL TERMS TO SUPPLEMENT Arber 2017 Utilities filter] | 125691 |
| 30 | 28 or 29 | 413495 |
| 31 | 11 and 30 [(m)CRPC & MEDLINE Utilities filter Arber2017 - balanced] | 838 |
| **32** | **31 use ppez [MEDLINE records]** | **145** |
| 33 | exp prostate cancer/ or (((prostate or prostatic) adj3 (adenocarcinoma$ or adeno-carcinoma$ or cancer$ or carcinoma$ or malignan$ or neoplas$ or tumor? or tumour?)) or PC or PCa).ti,ab,kw. | 715220 |
| 34 | orchiectomy/ and (insensitiv$ or refractor$ or resistan$).ti,ab,kw. | 2340 |
| 35 | (((androgen? or castrat$ or hormon$) adj2 (independen$ or insensitiv$ or refractor$ or resistan$)) or ((orchectom$ or orcheotom$ or orchidectom$ or orchiectom$ or testectom$ or (removal adj3 (testicle? or test#s))) and (insensitiv$ or refractor$ or resistan$))).ti,ab,kw. | 64389 |
| 36 | 33 and (34 or 35) | 47966 |
| 37 | castration resistant prostate cancer/ or (MCRPC or CRPC or MCRPCa or CRPCa or (((prostate or prostatic) adj3 (adenocarcinoma$ or adeno-carcinoma$ or cancer$ or carcinoma$ or malignan$ or neoplas$ or tumor? or tumour?)) and ((androgen? or castrat$ or hormon$) adj2 (independen$ or insensitiv$ or refractor$ or resistan$)))).ti,ab,kw. | 51418 |
| 38 | 36 or 37 | 51758 |
| 39 | exp animal/ or exp animal experimentation/ or exp animal model/ or exp animal experiment/ or nonhuman/ or exp vertebrate/ | 54437936 |
| 40 | exp human/ or exp human experimentation/ or exp human experiment/ | 42828307 |
| 41 | 39 not 40 | 11611361 |
| 42 | 38 not 41 [ANIMAL-ONLY REMOVED] | 49501 |
| 43 | editorial.pt. | 1277486 |
| 44 | letter.pt. not (letter.pt. and randomized controlled trial/) | 2330598 |
| 45 | 42 not (43 or 44) [OPINION PIECES REMOVED] | 48271 |
| 46 | Quality-Adjusted Life Year/ | 44522 |
| 47 | (quality adjusted or adjusted life year$).ti,ab,kw. | 52876 |
| 48 | (qaly$ or qald$ or qale$ or qtime$).ti,ab,kw. | 39243 |
| 49 | (illness state$1 or health state$1).ti,ab,kw. | 21068 |
| 50 | (hui or hui1 or hui2 or hui3).ti,ab,kw. | 4590 |
| 51 | (multiattribute$ or multi attribute$).ti,ab,kw. | 2384 |
| 52 | (utility adj3 (score$1 or valu$ or health$ or cost$ or measur$ or disease$ or mean or gain or gains or index$)).ti,ab,kw. | 48812 |
| 53 | utilities.ti,ab,kw. | 22153 |
| 54 | (eq-5d or eq5d or eq-5 or eq5 or euro qual or euroqual or euro qual5d or euroqual5d or euro qol or euroqol or euro qol5d or euroqol5d or euro quol or euroquol or euro quol5d or euroquol5d or eur qol or eurqol or eur qol5d or eur qol5d or eur?qul or eur?qul5d or euro$ quality of life or European qol).ti,ab,kw. | 49429 |
| 55 | (euro$ adj3 (5 d or 5d or 5 dimension$ or 5dimension$ or 5 domain$ or 5domain$)).ti,ab,kw. | 15545 |
| 56 | (sf36$ or sf 36$ or sf thirtysix or sf thirty six).ti,ab,kw. | 77721 |
| 57 | (time trade off$1 or time tradeoff$1 or tto or timetradeoff$1).ti,ab,kw. | 5390 |
| 58 | "quality of life"/ and ((quality of life or qol) adj (score$1 or measure$1)).ti,ab,kw. | 44056 |
| 59 | "quality of life"/ and ec.fs. | 59831 |
| 60 | "quality of life"/ and (health adj3 status).ti,ab,kw. | 28932 |
| 61 | (quality of life or qol).ti,ab,kw. and "cost benefit analysis"/ | 22075 |
| 62 | or/46-61 [Arber 2017 Utilities filter - balanced] | 350114 |
| 63 | exp "European Quality of Life 5 Dimensions questionnaire"/ or exp "Short Form 36"/ or ("European Quality of Life questionnaire 5D" or "disability adjusted life" or "sickness impact profile" or daly$ or (short form 36 or shortform 36 or shortform thirtysix or shortform thirty six or short form thirtysix or short form thirty six) or (sf6 or sf 6 or short form 6 or shortform 6 or sf six or sfsix or shortform six or short form six) or (sf12 or sf 12 or short form 12 or shortform 12 or sf twelve or sftwelve or shortform twelve or short form twelve) or (sf6D or sf 6D or short form 6D or shortform 6D or sf six D or sfsixD or shortform six D or short form six D) or (sf20 or sf 20 or short form 20 or shortform 20 or sf twenty or sftwenty or shortform twenty or short form twenty) or (hql or hqol or h qol or hrqol or hr qol) or (hye or hyes) or health$ year$ equivalent$ or disutili$ or rosser or (quality adj2 wellbeing) or qwb or standard gamble$).ti,ab. or (SG and gamble).ab. [ADDITIONAL TERMS TO SUPPLEMENT Arber 2017 Utilities filter] | 151606 |
| 64 | 62 or 63 | 422881 |
| 65 | 45 and 64 [(m)CRPC & Embase Utilities filter Arber2017 - balanced] | 1243 |
| 66 | conference abstract.pt. | 4172283 |
| 67 | 65 not 66 [CONFERENCE ABSTRACTS REMOVED] | 1004 |
| 68 | 65 and 66 | 239 |
| 69 | limit 68 to yr="2016 -Current" [MOST RECENT 5 YEARS CONF ABSTRACTS RETAINED] | 127 |
| 70 | 67 or 69 | 1131 |
| **71** | **70 use oemezd [Embase results]** | **856** |
| 72 | Prostatic Neoplasms/ or (((prostate or prostatic) adj3 (adenocarcinoma$ or adeno-carcinoma$ or cancer$ or carcinoma$ or malignan$ or neoplas$ or tumor? or tumour?)) or PC or PCa).ti,ab,kw. | 669701 |
| 73 | Orchiectomy/ and (insensitiv$ or refractor$ or resistan$).ti,ab,kw. | 2340 |
| 74 | (((androgen? or castrat$ or hormon$) adj2 (independen$ or insensitiv$ or refractor$ or resistan$)) or ((orchectom$ or orcheotom$ or orchidectom$ or orchiectom$ or testectom$ or (removal adj3 (testicle? or test#s))) and (insensitiv$ or refractor$ or resistan$))).ti,ab,kw. | 64389 |
| 75 | 72 and (73 or 74) | 47895 |
| 76 | Prostatic Neoplasms, Castration-Resistant/ or (MCRPC or CRPC or MCRPCa or CRPCa or (((prostate or prostatic) adj3 (adenocarcinoma$ or adeno-carcinoma$ or cancer$ or carcinoma$ or malignan$ or neoplas$ or tumor? or tumour?)) and ((androgen? or castrat$ or hormon$) adj2 (independen$ or insensitiv$ or refractor$ or resistan$)))).ti,ab,kw. | 50770 |
| 77 | 75 or 76 | 51052 |
| 78 | Quality-Adjusted Life Years/ | 44522 |
| 79 | (quality adjusted or adjusted life year$).mp. | 68761 |
| 80 | (qaly$ or qald$ or qale$ or qtime$).mp. | 39273 |
| 81 | (illness state$1 or health state$1).mp. | 21125 |
| 82 | (hui or hui1 or hui2 or hui3).mp. | 5507 |
| 83 | (multiattribute$ or multi attribute$).mp. | 2405 |
| 84 | (utility adj3 (score$1 or valu$ or health$ or cost$ or measur$ or disease$ or mean or gain or gains or index$)).mp. | 53030 |
| 85 | utilities.mp. | 22244 |
| 86 | (eq-5d or eq5d or eq-5 or eq5 or euro qual or euroqual or euro qual5d or euroqual5d or euro qol or euroqol or euro qol5d or euroqol5d or euro quol or euroquol or euro quol5d or euroquol5d or eur qol or eurqol or eur qol5d or eur qol5d or eur?qul or eur?qul5d or euro$ quality of life or European qol).mp. | 51115 |
| 87 | (euro$ adj3 (5 d or 5d or 5 dimension$ or 5dimension$ or 5 domain$ or 5domain$)).mp. | 16990 |
| 88 | (sf36$ or sf 36$ or sf thirtysix or sf thirty six).mp. | 77755 |
| 89 | (time trade off$1 or time tradeoff$1 or tto or timetradeoff$1).mp. | 5417 |
| 90 | quality of life/ and ((quality of life or qol) adj (score$1 or measure$1)).mp. | 44140 |
| 91 | quality of life/ and ec.fs. | 59831 |
| 92 | quality of life/ and (health adj3 status).mp. | 63427 |
| 93 | Cost-Benefit Analysis/ and (quality of life or qol).mp. | 30522 |
| 94 | or/78-93 [Arber 2017 Utilities filter - balanced] | 379965 |
| 95 | ("European Quality of Life questionnaire 5D" or "disability adjusted life" or "sickness impact profile" or daly$ or (short form 36 or shortform 36 or shortform thirtysix or shortform thirty six or short form thirtysix or short form thirty six) or (sf6 or sf 6 or short form 6 or shortform 6 or sf six or sfsix or shortform six or short form six) or (sf12 or sf 12 or short form 12 or shortform 12 or sf twelve or sftwelve or shortform twelve or short form twelve) or (sf6D or sf 6D or short form 6D or shortform 6D or sf six D or sfsixD or shortform six D or short form six D) or (sf20 or sf 20 or short form 20 or shortform 20 or sf twenty or sftwenty or shortform twenty or short form twenty) or (hql or hqol or h qol or hrqol or hr qol) or (hye or hyes) or health$ year$ equivalent$ or disutili$ or rosser or (quality adj2 wellbeing) or qwb or standard gamble$).ti,ab. or (SG and gamble).ab. [ADDITIONAL TERMS TO SUPPLEMENT Arber 2017 Utilities filter] | 125691 |
| 96 | 94 or 95 | 440426 |
| 97 | 77 and 96 [(m)CRPC & CENTRAL Utilities filter Arber2017 - balanced] | 1252 |
| **98** | **97 use cctr** | **142** |
| 99 | 32 or 71 or 98 | 1143 |
| **100** | **remove duplicates from 99 [All databases - deduplicated]** | **955** |

Table S3: Search Strategy (October 3, 2022)

| **#** | **Searches** | **Results** |
| --- | --- | --- |
| 1 | Prostatic Neoplasms/ or (((prostate or prostatic) adj3 (adenocarcinoma$ or adeno-carcinoma$ or cancer$ or carcinoma$ or malignan$ or neoplas$ or tumor? or tumour?)) or PC or PCa).ti,ab,kf. | 715780 |
| 2 | Orchiectomy/ and (insensitiv$ or refractor$ or resistan$).ti,ab,kf. | 2434 |
| 3 | (((androgen? or castrat$ or hormon$) adj2 (independen$ or insensitiv$ or refractor$ or resistan$)) or ((orchectom$ or orcheotom$ or orchidectom$ or orchiectom$ or testectom$ or (removal adj3 (testicle? or test#s))) and (insensitiv$ or refractor$ or resistan$))).ti,ab,kf. | 69106 |
| 4 | 1 and (2 or 3) | 52075 |
| 5 | Prostatic Neoplasms, Castration-Resistant/ or (MCRPC or CRPC or MCRPCa or CRPCa or (((prostate or prostatic) adj3 (adenocarcinoma$ or adeno-carcinoma$ or cancer$ or carcinoma$ or malignan$ or neoplas$ or tumor? or tumour?)) and ((androgen? or castrat$ or hormon$) adj2 (independen$ or insensitiv$ or refractor$ or resistan$)))).ti,ab,kf. | 55317 |
| 6 | 4 or 5 | 55604 |
| 7 | exp Animals/ not Humans/ | 16530187 |
| 8 | 6 not 7 [ANIMAL-ONLY REMOVED] | 43438 |
| 9 | (comment or editorial or news or newspaper article).pt. | 2362415 |
| 10 | (letter not (letter and randomized controlled trial)).pt. | 2432024 |
| 11 | 8 not (9 or 10) [OPINION PIECES REMOVED] | 41918 |
| 12 | Quality-Adjusted Life Years/ | 49151 |
| 13 | (quality adjusted or adjusted life year$).ti,ab,kf. | 58870 |
| 14 | (qaly$ or qald$ or qale$ or qtime$).ti,ab,kf. | 43447 |
| 15 | (illness state$1 or health state$1).ti,ab,kf. | 22978 |
| 16 | (hui or hui1 or hui2 or hui3).ti,ab,kf. | 5044 |
| 17 | (multiattribute$ or multi attribute$).ti,ab,kf. | 2709 |
| 18 | (utility adj3 (score$1 or valu$ or health$ or cost$ or measur$ or disease$ or mean or gain or gains or index$)).ti,ab,kf. | 53035 |
| 19 | utilities.ti,ab,kf. | 24301 |
| 20 | (eq-5d or eq5d or eq-5 or eq5 or euro qual or euroqual or euro qual5d or euroqual5d or euro qol or euroqol or euro qol5d or euroqol5d or euro quol or euroquol or euro quol5d or euroquol5d or eur qol or eurqol or eur qol5d or eur qol5d or eur?qul or eur?qul5d or euro$ quality of life or European qol).ti,ab,kf. | 56205 |
| 21 | (euro$ adj3 (5 d or 5d or 5 dimension$ or 5dimension$ or 5 domain$ or 5domain$)).ti,ab,kf. | 17259 |
| 22 | (sf36$ or sf 36$ or sf thirtysix or sf thirty six).ti,ab,kf. | 82888 |
| 23 | (time trade off$1 or time tradeoff$1 or tto or timetradeoff$1).ti,ab,kf. | 5856 |
| 24 | quality of life/ and ((quality of life or qol) adj (score$1 or measure$1)).ti,ab,kf. | 48239 |
| 25 | quality of life/ and ec.fs. | 66875 |
| 26 | quality of life/ and (health adj3 status).ti,ab,kf. | 31879 |
| 27 | (quality of life or qol).ti,ab,kf. and Cost-Benefit Analysis/ | 25659 |
| 28 | or/12-27 [Arber 2017 Utilities filter - balanced] | 385077 |
| 29 | ("European Quality of Life questionnaire 5D" or "disability adjusted life" or "sickness impact profile" or daly$ or (short form 36 or shortform 36 or shortform thirtysix or shortform thirty six or short form thirtysix or short form thirty six) or (sf6 or sf 6 or short form 6 or shortform 6 or sf six or sfsix or shortform six or short form six) or (sf12 or sf 12 or short form 12 or shortform 12 or sf twelve or sftwelve or shortform twelve or short form twelve) or (sf6D or sf 6D or short form 6D or shortform 6D or sf six D or sfsixD or shortform six D or short form six D) or (sf20 or sf 20 or short form 20 or shortform 20 or sf twenty or sftwenty or shortform twenty or short form twenty) or (hql or hqol or h qol or hrqol or hr qol) or (hye or hyes) or health$ year$ equivalent$ or disutili$ or rosser or (quality adj2 wellbeing) or qwb or standard gamble$).ti,ab. or (SG and gamble).ab. [ADDITIONAL TERMS TO SUPPLEMENT Arber 2017 Utilities filter] | 138187 |
| 30 | 28 or 29 | 456590 |
| 31 | 11 and 30 [(m)CRPC & MEDLINE Utilities filter Arber2017 - balanced] | 973 |
| 32 | 31 use ppez [MEDLINE records] | 175 |
| 33 | exp prostate cancer/ or (((prostate or prostatic) adj3 (adenocarcinoma$ or adeno-carcinoma$ or cancer$ or carcinoma$ or malignan$ or neoplas$ or tumor? or tumour?)) or PC or PCa).ti,ab,kw. | 765268 |
| 34 | orchiectomy/ and (insensitiv$ or refractor$ or resistan$).ti,ab,kw. | 2418 |
| 35 | (((androgen? or castrat$ or hormon$) adj2 (independen$ or insensitiv$ or refractor$ or resistan$)) or ((orchectom$ or orcheotom$ or orchidectom$ or orchiectom$ or testectom$ or (removal adj3 (testicle? or test#s))) and (insensitiv$ or refractor$ or resistan$))).ti,ab,kw. | 68369 |
| 36 | 33 and (34 or 35) | 51655 |
| 37 | castration resistant prostate cancer/ or (MCRPC or CRPC or MCRPCa or CRPCa or (((prostate or prostatic) adj3 (adenocarcinoma$ or adeno-carcinoma$ or cancer$ or carcinoma$ or malignan$ or neoplas$ or tumor? or tumour?)) and ((androgen? or castrat$ or hormon$) adj2 (independen$ or insensitiv$ or refractor$ or resistan$)))).ti,ab,kw. | 55569 |
| 38 | 36 or 37 | 55940 |
| 39 | exp animal/ or exp animal experimentation/ or exp animal model/ or exp animal experiment/ or nonhuman/ or exp vertebrate/ | 57650014 |
| 40 | exp human/ or exp human experimentation/ or exp human experiment/ | 45592256 |
| 41 | 39 not 40 | 12059588 |
| 42 | 38 not 41 [ANIMAL-ONLY REMOVED] | 53605 |
| 43 | editorial.pt. | 1360756 |
| 44 | letter.pt. not (letter.pt. and randomized controlled trial/) | 2431964 |
| 45 | 42 not (43 or 44) [OPINION PIECES REMOVED] | 52304 |
| 46 | Quality-Adjusted Life Year/ | 49151 |
| 47 | (quality adjusted or adjusted life year$).ti,ab,kw. | 58772 |
| 48 | (qaly$ or qald$ or qale$ or qtime$).ti,ab,kw. | 43365 |
| 49 | (illness state$1 or health state$1).ti,ab,kw. | 22858 |
| 50 | (hui or hui1 or hui2 or hui3).ti,ab,kw. | 5022 |
| 51 | (multiattribute$ or multi attribute$).ti,ab,kw. | 2701 |
| 52 | (utility adj3 (score$1 or valu$ or health$ or cost$ or measur$ or disease$ or mean or gain or gains or index$)).ti,ab,kw. | 52363 |
| 53 | utilities.ti,ab,kw. | 24153 |
| 54 | (eq-5d or eq5d or eq-5 or eq5 or euro qual or euroqual or euro qual5d or euroqual5d or euro qol or euroqol or euro qol5d or euroqol5d or euro quol or euroquol or euro quol5d or euroquol5d or eur qol or eurqol or eur qol5d or eur qol5d or eur?qul or eur?qul5d or euro$ quality of life or European qol).ti,ab,kw. | 56465 |
| 55 | (euro$ adj3 (5 d or 5d or 5 dimension$ or 5dimension$ or 5 domain$ or 5domain$)).ti,ab,kw. | 17193 |
| 56 | (sf36$ or sf 36$ or sf thirtysix or sf thirty six).ti,ab,kw. | 83090 |
| 57 | (time trade off$1 or time tradeoff$1 or tto or timetradeoff$1).ti,ab,kw. | 5835 |
| 58 | "quality of life"/ and ((quality of life or qol) adj (score$1 or measure$1)).ti,ab,kw. | 48188 |
| 59 | "quality of life"/ and ec.fs. | 66875 |
| 60 | "quality of life"/ and (health adj3 status).ti,ab,kw. | 31111 |
| 61 | (quality of life or qol).ti,ab,kw. and "cost benefit analysis"/ | 25671 |
| 62 | or/46-61 [Arber 2017 Utilities filter - balanced] | 384880 |
| 63 | exp "European Quality of Life 5 Dimensions questionnaire"/ or exp "Short Form 36"/ or ("European Quality of Life questionnaire 5D" or "disability adjusted life" or "sickness impact profile" or daly$ or (short form 36 or shortform 36 or shortform thirtysix or shortform thirty six or short form thirtysix or short form thirty six) or (sf6 or sf 6 or short form 6 or shortform 6 or sf six or sfsix or shortform six or short form six) or (sf12 or sf 12 or short form 12 or shortform 12 or sf twelve or sftwelve or shortform twelve or short form twelve) or (sf6D or sf 6D or short form 6D or shortform 6D or sf six D or sfsixD or shortform six D or short form six D) or (sf20 or sf 20 or short form 20 or shortform 20 or sf twenty or sftwenty or shortform twenty or short form twenty) or (hql or hqol or h qol or hrqol or hr qol) or (hye or hyes) or health$ year$ equivalent$ or disutili$ or rosser or (quality adj2 wellbeing) or qwb or standard gamble$).ti,ab. or (SG and gamble).ab. [ADDITIONAL TERMS TO SUPPLEMENT Arber 2017 Utilities filter] | 169618 |
| 64 | 62 or 63 | 466050 |
| 65 | 45 and 64 [(m)CRPC & Embase Utilities filter Arber2017 - balanced] | 1375 |
| 66 | conference abstract.pt. | 4561000 |
| 67 | 65 not 66 [CONFERENCE ABSTRACTS REMOVED] | 1113 |
| 68 | 65 and 66 | 262 |
| 69 | limit 68 to yr="2016 -Current" [MOST RECENT 5 YEARS CONF ABSTRACTS RETAINED] | 150 |
| 70 | 67 or 69 | 1263 |
| 71 | 70 use oemezd [Embase results] | 949 |
| 72 | Prostatic Neoplasms/ or (((prostate or prostatic) adj3 (adenocarcinoma$ or adeno-carcinoma$ or cancer$ or carcinoma$ or malignan$ or neoplas$ or tumor? or tumour?)) or PC or PCa).ti,ab,kw. | 713264 |
| 73 | Orchiectomy/ and (insensitiv$ or refractor$ or resistan$).ti,ab,kw. | 2418 |
| 74 | (((androgen? or castrat$ or hormon$) adj2 (independen$ or insensitiv$ or refractor$ or resistan$)) or ((orchectom$ or orcheotom$ or orchidectom$ or orchiectom$ or testectom$ or (removal adj3 (testicle? or test#s))) and (insensitiv$ or refractor$ or resistan$))).ti,ab,kw. | 68369 |
| 75 | 72 and (73 or 74) | 51563 |
| 76 | Prostatic Neoplasms, Castration-Resistant/ or (MCRPC or CRPC or MCRPCa or CRPCa or (((prostate or prostatic) adj3 (adenocarcinoma$ or adeno-carcinoma$ or cancer$ or carcinoma$ or malignan$ or neoplas$ or tumor? or tumour?)) and ((androgen? or castrat$ or hormon$) adj2 (independen$ or insensitiv$ or refractor$ or resistan$)))).ti,ab,kw. | 54884 |
| 77 | 75 or 76 | 55182 |
| 78 | Quality-Adjusted Life Years/ | 49151 |
| 79 | (quality adjusted or adjusted life year$).mp. | 76341 |
| 80 | (qaly$ or qald$ or qale$ or qtime$).mp. | 43451 |
| 81 | (illness state$1 or health state$1).mp. | 22992 |
| 82 | (hui or hui1 or hui2 or hui3).mp. | 5982 |
| 83 | (multiattribute$ or multi attribute$).mp. | 2735 |
| 84 | (utility adj3 (score$1 or valu$ or health$ or cost$ or measur$ or disease$ or mean or gain or gains or index$)).mp. | 57608 |
| 85 | utilities.mp. | 24358 |
| 86 | (eq-5d or eq5d or eq-5 or eq5 or euro qual or euroqual or euro qual5d or euroqual5d or euro qol or euroqol or euro qol5d or euroqol5d or euro quol or euroquol or euro quol5d or euroquol5d or eur qol or eurqol or eur qol5d or eur qol5d or eur?qul or eur?qul5d or euro$ quality of life or European qol).mp. | 59125 |
| 87 | (euro$ adj3 (5 d or 5d or 5 dimension$ or 5dimension$ or 5 domain$ or 5domain$)).mp. | 17605 |
| 88 | (sf36$ or sf 36$ or sf thirtysix or sf thirty six).mp. | 83176 |
| 89 | (time trade off$1 or time tradeoff$1 or tto or timetradeoff$1).mp. | 5887 |
| 90 | quality of life/ and ((quality of life or qol) adj (score$1 or measure$1)).mp. | 48332 |
| 91 | quality of life/ and ec.fs. | 66875 |
| 92 | quality of life/ and (health adj3 status).mp. | 67681 |
| 93 | Cost-Benefit Analysis/ and (quality of life or qol).mp. | 34498 |
| 94 | or/78-93 [Arber 2017 Utilities filter - balanced] | 417513 |
| 95 | ("European Quality of Life questionnaire 5D" or "disability adjusted life" or "sickness impact profile" or daly$ or (short form 36 or shortform 36 or shortform thirtysix or shortform thirty six or short form thirtysix or short form thirty six) or (sf6 or sf 6 or short form 6 or shortform 6 or sf six or sfsix or shortform six or short form six) or (sf12 or sf 12 or short form 12 or shortform 12 or sf twelve or sftwelve or shortform twelve or short form twelve) or (sf6D or sf 6D or short form 6D or shortform 6D or sf six D or sfsixD or shortform six D or short form six D) or (sf20 or sf 20 or short form 20 or shortform 20 or sf twenty or sftwenty or shortform twenty or short form twenty) or (hql or hqol or h qol or hrqol or hr qol) or (hye or hyes) or health$ year$ equivalent$ or disutili$ or rosser or (quality adj2 wellbeing) or qwb or standard gamble$).ti,ab. or (SG and gamble).ab. [ADDITIONAL TERMS TO SUPPLEMENT Arber 2017 Utilities filter] | 138187 |
| 96 | 94 or 95 | 484246 |
| 97 | 77 and 96 [(m)CRPC & CENTRAL Utilities filter Arber2017 - balanced] | 1380 |
| 98 | 97 use cctr | 154 |
| 99 | 32 or 71 or 98 | 1278 |
| 100 | limit 32 to dt="20210801-20221231" [MEDLINE update: Aug 2021-Current] | 25 |
| 101 | limit 71 to dd="20210801-20221231" [Embase update: Aug 2021-Current] | 33 |
| 102 | 98 and (202107$ or 202108$ or 202109$ or 202110$ or 202111$ or 202112$ or 2022$).up. [CENTRAL update: Jul 2021-Current] | 120 |
| 103 | 100 or 101 or 102 [All databases - update: Jul/Aug 2021-Current] | 178 |
| 104 | limit 103 to yr="2021 -Current" [All database results update: Jul/Aug 2021-Current - 2021 & 2022 publication year limit] | 76 |
| **105** | **remove duplicates from 104 [All databases - deduplicated]** | **63** |

Table S4: List of Congresses Manually Searched

| **Conference Searches** | **Hand Searching** |
| --- | --- |
| Key conferences from 2019-2022 were searched to identify relevant abstracts:   - American Association for Cancer Research (AACR) - American Society of Clinical Oncology (ASCO) - American Society of Clinical Oncology Genitourinary Cancers Symposium (ASCO-GU) - American Urological Association (AUA) - European Association of Urology (EAU) - European Cancer Organization (ECCO) - European Multidisciplinary Congress on Urological Cancers (EMUC) - European Society of Medical Oncology (ESMO) - EAU Section of Oncological Urology (ESOU) - National Comprehensive Cancer Network (NCCN) - Society of Urologic Oncology (SUO) - International Society for Pharmacoeconomics and Outcomes Research (ISPOR) - International Society for Pharmacoeconomics and Outcomes Research Europe (ISPOR-EU) | HTA bodies and other databases were searched to identify:   - We performed targeted searches of HTA and regulatory bodies to identify additional studies of interest:   - Food and Drug Administration (FDA)   - European Medicines Agency (EMA)   - Canadian Agency for Drugs and Technologies in Health (CADTH)   - National Institute for Health and Care Excellence (NICE)   - Scottish Medicines Agency (SMC) - Additionally, we performed searches of:   - Tuft Medical Center Cost-Effectiveness Analysis Registry   - Bibliographies of relevant SLRs captured from the database searches - Health Economics Research Centre (HERC) database of mapping studies - Center for Disease Control (CDC) HRQoL - School of Health and Related Research Health Utility Database (ScHARRHUD) |

Table S5: List of Included Studies Reporting on Utility Values or VAS Scores

| **Author, Year/ID #, Author, Year^A^** | **Study Design** | **Location** | **Outcomes Reported** |
| --- | --- | --- | --- |
| Pu, 2022 | Phase 3 RCT | East Asia | EQ VAS |
| Heidenreich, 2017 | Phase 2 RCT | Global | EQ-5D-5L  EQ VAS |
| Loriot, 2015  Devlin, 2017 | Phase 3 RCT | Global | EQ-5D  EQ VAS |
| Saad, 2017 | Subgroup Analysis | Global | EQ-5D |
| Lloyd, 2015 | Cross-Sectional Survey | UK | EQ-5D-5L  EQ VAS |
| Diels, 2015 | Cross-sectional Observational Study | Europe | EQ-5D |
| Payne, 2021 | Prospective, Observational Study | Europe | EQ-5D-5L |
| Dearden, 2019 | Cross-Sectional Survey with Semi-Structured Interviews | UK, France, Germany | EQ-5D-5L  EQ VAS |
| Procopio, 2020  Procopio, 2022 | Prospective, Observational Study | Italy | EQ-5D-3L  EQ VAS |
| Bahl, 2015 | Single-arm Trial | UK | EQ-5D-3L |
| Kramer, 2020  Fizazi, 2020 | Randomized, Open Label Trial | Global | EQ-5D-5L  EQ VAS |
| Murasawa, 2019 | Cross-Sectional Observational Study | Japan | EQ-5D-5L  EQ VAS |
| Saad, 2022 | Phase 2 Trial | Global | EQ VAS |
| Saad, 2022 | Single-arm Trial | Global | EQ-5D-5L  EQ VAS |
| Boye, 2022 | Real-World Study | Global | EQ-5D-5L  EQ VAS |
| Kuppen, 2020 | Prospective, Observational Study | Netherlands | EQ-5D  EQ VAS |
| Parente, 2017 | Observational Study | Australia | AQoL |
| Skaltsa, 2014 | Utility Mapping | Global | FACT-P mapped to EQ-5D |
| Wu, 2007 | Utility Mapping | Global | QLQ-C30 mapped to EQ-5D |
| Kim, 2022 | Utility Weights Estimate | South Korea | Standard Gamble |
| Gong, 2014 | CUA | US | EQ-5D HSUV |
| Hill, 2019 | CUA | Canada | EQ-5D HSUV |
| Okumura, 2021 | CUA | Japan | EQ-5D HSUV |
| Zhang, 2021 | CUA | US | EQ-5D HSUV |
| Ten Ham, 2021 | CUA | Netherlands | EQ-5D HSUV |
| Ko, 2021 | CUA | US | EQ-5D HSUV |
| Su, 2020 | CUA | US | EQ-5D HSUV |
| Barqawi, 2019 | CUA | US | EQ-5D HSUV |
| Sanyal, 2016 | CUA | Canada | EQ-5D HSUV |
| Wilson, 2014 | CUA | US | QWB HSUV |
| Holko, 2014 | CUA | US | EQ-5D HSUV |
| Zhong, 2013 | CUA | US | QWB HSUV |
| Li, 2021 | CUA | US | EQ-5D HSUV |
| Xu, 2022 | CUA | US & China | EQ-5D HSUV |
| Sanyal, 2014 | CUA with model validation | Canada | EQ-5D HSUV |
| 485-NICE-2016  486-NICE-2016 | HTA Submission | UK | FACT-P mapped to EQ-5D |
| 482-NICE-2015  483-NICE-2016 | HTA Submission | UK | EQ-5D HSUV |
| Collins, 2007 | HTA Submission with Therapeutic Review | UK | EQ-5D HSUV |
| 629-SMC-2015 | HTA Submission | Scotland | EQ-5D HSUV |
| 439-NICE-2020 | HTA Submission | UK | EQ-5D-5L mapped to EQ-5D-3L HSUVs |
| 468-NICE-2012  469-NICE-2012 | HTA Submission | UK | FACT-P mapped to EQ-5D |
| 470-NICE-2014  471-NICE-2014 | HTA Submission | UK | FACT-P mapped to EQ-5D |
| 488-NICE-2016  489-NICE-2016 | HTA Submission | UK | EQ-5D HSUV |
| 620-SMC-2016 | HTA Submission | Scotland | EQ-5D HSUV |
| 633-SMC-2013 | HTA Submission | Scotland | EQ-5D HSUV |
| 667-SMC-2022 | HTA Submission | Scotland | EQ-5D-5L mapped to EQ-5D-3L HSUVs |

^A^ To avoid confusion of HTA submissions from the same year, the identifying reference number was included

Abbreviations: AQoL = Assessment of Quality of Life; CUA = cost-utility analysis; EQ-5D = EuroQoL 5-Dimension; FACT-P = Functional Assessment of Cancer Therapy – Prostate; HSUV = heath state utility value; HTA = health technology assessment; NICE = National Institute for Health and Care Excellence; QWB = Quality of Well-being; RCT = randomized controlled trial; SMC = Scottish Medicines Agency; VAS = visual analog scale; UK = United Kingdom; US = United States of America

Table S6: Quality Assessment – Primary Studies

| **Trial;NCT**  **Reference** | **Sample Size** | **Respondent Selection and Recruitment** | **Inclusion/Exclusion Criteria** | **Response Rates to Instruments Used** | **Loss to Follow-up** | **Missing Data** | **Any Other Problems With the Study** |
| --- | --- | --- | --- | --- | --- | --- | --- |
| ABItude  80-Procopio-2020 | Trial (Full Analysis): n=453 Utilities: n=453 | All eligible patients with mCRPC naïve to chemotherapy were consecutively enrolled in 49 Italian participating centers at the time of initiating abiraterone acetate plus prednisone therapy. All patients from the full analysis set were included in the assessment of QoL and pain. Only questionnaires filled in while the patient was under treatment with abiraterone were evaluated in the analyses. | Patients not eligible according to clinician’s declaration or Source Document Verification, those with missing date of diagnosis, and those with prior abiraterone treatment were excluded from the full analysis set. | Data for VAS were available from 431 patients at baseline, 329 at 6-month follow-up, 224 at 12-month follow-up, 139 at 18-month follow-up and 68 at 24-month follow-up | 285 patients permanently discontinued abiraterone, mainly because of disease progression (184 patients, 65% who discontinued abiraterone). Other reasons for treatment discontinuation included death (n = 27, 9.5%), personal choice (n = 17, 6%), adverse reaction (n = 14, 4.9%) and AEs (not drug-related) (n = 11, 3.9%) | Other missing data reported, however HRQoL- related data was not reported | The lack of a control arm prevents the comparisons of the effectiveness of abiraterone to that of other first-line treatments for mCRPC, including chemotherapy and other AR-directed agents |
| NR  191-Dearden-2019 | Interviews: n=38 Surverys: n=152 | Clinicians in each country (France, Germany, and the UK) identified eligible patients | The data from all patients were entered into a database for analysis. eligible patients had to have been receiving abiraterone or enzalutamide treatment for a minimum of two months | Data not provided. | NA; study was cross-sectional | Data not provided. | The UK sample was notable smaller when compared to the number of patients recruited from France and Germany, which may affect the generalizability of the results to the UK. The cross-sectional design may bias results as patients were only assessed at a single timepoint |
| NCT01080352  348-Nielson-2017 | Enrolled: n=23 Evaluated: n=20 | Single-centre; patients were recruited from the outpatient urology clinic at Copenhagen University Hospital Herlev | The key exclusion criteria were surgical removal or radiation of the prostate; significant renal impairment; significant cardiac disease; history of oxalate renal stones; hemochromatosis; and glucose-6-phosphate dehydrogenase deficiency | Data not provided. | Discontinued before week 12: due to disease progression, n=2 due to perceived side effects, n=1  Discontinued follow-up before week 20, n=5 Discontinued follow-up before week 26, n=1 Discontinued follow-up before week 52, n=8 | Other missing data reported, however HRQoL- related data was not reported | Single-centre design may effect the generalizability of the results. Single arm design study which limits the strength of the implications that can be drawn from the analysis. Small sample size. |
| NR  477-Lloyd-2015 | Survey: n=50 | Eligible participants were recruited through a specialist patient recruitment agency, via Prostate Cancer Support Federation support groups, and through the Cancer Research UK Web site. | Patients were excluded if they had brain metastases, chronic liver disease, serious liver impairment, and/or viral hepatitis due to potential significant confounding impact on HRQoL | Data not provided. | NA; study was cross-sectional | The online survey requested that participants complete all items to progress through the survey, which meant there was no missing data. | Patients received £45 or reward points for completion of the survey. This study was cross-sectional and does not capture longterm changes in HRQoL. Online survey methodology may be biased to younger/healthier participants. Small sample size. |
| NR  495-Diels-2015 | Study: n=602 Utilities (chemotherapy naïve): n=236 | HRQoL data were collected from patients with mCRPC who were enrolled in an observational study conducted in 47 centers across six European Union countries. Consecutive patients who visited the clinic during regular follow-up visits were invited to participate | Exclusion criteria included participation in any investigational drug study or any expanded access program during the observation period. | Complete FACT-P and EQ-5D questionnaires were available for 602 (86%) patients | NA; study was cross-sectional | Data not provided. | This study used cross-sectional data and was unable to analyze longterm quality of life trends. |
| PREMISE: NCT02495974  17U-Payne-2021 | Trial: n = 1732 Cohort 1 (chemo-naïve and abi-naïve): n = 1171 EQ index: 1109 EQ VAS: 1105 FACT-P: 1086 FACT-G: 1090 FACT-PCS: 1103 BPI- SF (severity): 1075 BPI-SF (interference): 1065 | Patients were recruited from 182 study locations in Europe. Eligible patients were men with mCRPC who were prescribed enzalutamide as a part of routine clinical practice. Patients completed PRO assessments to fulfil secondary endpoint. | Patients with a history of abiraterone prior to chemotherapy were not included in the study. Patients who had received any other (non-docetaxel) chemotherapeutic agents were not included in the study | In the enza cohort 1 (chemo-naïve and abi-naïve) at the last reported timepoint (month 9) was around 50%: EQ index: 50.3% (558/1109) EQ VAS: 50.5% (558/1105) FACT-P: 50% (543/1086) FACT-G: 50.5% (550/1090) FACT-PCS: 50.6% (558/1103) BPI- SF (severity): 49.5% (532/1075) BPI-SF (interference): 48.7% (519/1065) | 109/1175 (9.3%) of patients were lost to follow-up between baseline and end of study. | Due to follow-up time limitations, many patients were censored, mainly in Cohort 1. For PROs, data points after 9 months were not reported due to patient dropout. | Due to the study's observational, nonrandomized design, data interpretation is limited due to lack of a comparative control group and formal statistical comparisons. Another limitation is potential selection bias, as recruitment at specific study sites may not be representative of the larger population and patients were not randomly assigned to enzalutamide treatment |
| PRO-CAPRI: NL3934  96-Kuppen-2020 | Trial: n = 167  Baseline questionnaires returned: = 151  CT-naïve: n =112  Post-CT: n = 39 | Eligible patients provided written informed consent to the treating physician at the hospital site. All PRO-CAPRI patients were also included in the CAPRI registry. Subgroups were created based on the disease state at inclusion, namely chemotherapy-naive state (CTx-naive [ie, no prior docetaxel treatment]) and (post-) chemotherapy state (post-CTx [ie, current docetaxel or post-docetaxel treatment]). | Patients diagnosed with mCRPC between January 1, 2010 and  December 31, 2015 were eligible for inclusion, conforming to the  CAPRI inclusion criteria.9 Patients were eligible for the PROCAPRI study from diagnosis of CRPC to 4 weeks after the start  of the first post-docetaxel treatment | The compliance rate ranged from 94% to 100% per questionnaire, except for BPI-SF, which was added during the study after a protocol amendment | Termination of the study before the maximum follow-up of 2 years occurred in 113 (75%) patients, owing to death (n = 56; 37%), lost-to-follow-up (n = 22; 15%), withdrawal of informed consent (n = 9; 6%), or database cutoff (n = 26; 17%) | No missing data was reported | An important limitation of this study was the relatively small sample size. Only 4 percent of all patients included in the CAPRIregistry were included in the PRO-CAPRI study. At baseline mCRPC diagnosis, patients in the PRO-CAPRI study tended to be in better clinical condition than patients in the CAPRI-registry. Therefore, results are possibly not generalizable for the total Dutch population. The second limitation of this study was the nonrandomized study design that made it impossible to compare the individual new treatments. Subgroups per treatment were too small for reliable analyses of changes in HRQoL |
| CARD: NCT02485691  115-Fizazi-2020 | Recruited: n = 255  Cabazitaxel: n = 129  Enzalutamide/Abiraterone: n = 126 | Patients were recruited from 62 sites in 13 European countries. Patients were aged ≥18 years, Eastern Cooperative Oncology Group (ECOG) performance status ≤2, with confirmed metastatic castration-resistant prostate cancer | Briefly, patients with prostate cancer were eligible if they had castrate amounts of testosterone had disease progression per Response Evaluation Criteria in Solid Tumors version 1.1, or had at least two new bone lesions or a rising prostate-specific antigen (PSA) concentration according to Prostate Cancer Working Group 2 criteria, had received at least three cycles of docetaxel, and had progressed within 12 months of androgen signallingtargeted inhibitor treatment. Use of abiraterone and docetaxel in metastatic hormone-sensitive disease was allowed. Exclusion criteria included age younger than 18 years, Eastern Cooperative Oncology Group (ECOG) performance status higher than 2, previous chemotherapy (except docetaxel), history of seizure, inadequate organ or bone marrow function, history of previous malignancy within 5 years, history of mineralocorticoid excess or deficiency, and uncontrolled severe illness or medical condition | Not reported | 2 patients were lost to follow-up in the cabazitaxel group, with 120 discontinuing treatment at some point (either because of disease progression, adverse events, investigator decision, or other). 117 discontinued in the enzalutamide or abiraterone group | No formal imputation for missing data was done, and reasons for missing data were not centrally recorded | CARD was an open label trial with a fairly small sample size, and was not powered for quality of life. Patients had also progressed last on a single therapy, meaning the patients may not be generalizable in the era of combination therapies. There are also inherent multiplicity issues with recording outcomes at multiple timepoints throughout treatment, and issues relating to lower patient numbers at the end of the study |
| 170-Murasawa-2019 | DM-CRPC patients: n = 38 | 100 patients were registered at each hospital (500 total), of which 380 patients data were analyzed. 38 of those patients had DM-CRPC. Only DM-CRPC patients were extracted | In each hospital, patients selected were≥20 years of age and pathologically diagnosed with PC. | Response rates were not available for DM-CRPC group | Not reported | Not reported | There may be bias in using EQ-5D-5L and not FACT-P since FACT-P is the prostate cancer-specific scale, however the focus of the study is on health utility.They also highlighted that there could be differences in how the registered hospitals recorded disease progression, as they had very few DM-CRPC patients |
| 329-Parente-2017 | mCRPC patients: n= 104 | The study population comprised patients receiving cabazitaxel through the Australian EAP. Patients were eligible for the EAP if they were aged at least 18 years, had confirmed mCRPC previously treated with a docetaxel-containing regimen and experienced disease progression during or after docetaxel treatment | Additional inclusion criteria were ongoing surgical or medical castration; Eastern Cooperative Oncology Group (ECOG) performance status (PS) 0, 1 or 2; life expectancy ≥3 months; adequate bone marrow, renal and liver function; and written informed consent. Exclusion criteria included prior radiotherapy to ≥40% of bone marrow; prior radionucleotide therapy with samarium-153, P-32 within 8 weeks prior to enrolment or with strontium-89 or radium-223 within 12 weeks prior to enrolment; prior surgery, radiation or chemotherapy within 4 weeks prior to enrolment; active grade ≥2 peripheral neuropathy; active grade ≥2 stomatitis; active infection requiring systemic antibiotic or anti-fungal medication; active cancer (other than mCRPC) including prior malignancy from which the patient has been disease-free for ≤5 years (except superficial nonmelanoma skin cancer); known brain or leptomeningeal involvement; history of severe hypersensitivity reaction (≥grade 3) to docetaxel; history of severe hypersensitivity reaction (≥grade 3) to polysorbate 80 containing drugs; history of severe hypersensitivity reaction (≥grade 3) or intolerance to prednisone or prednisolone; uncontrolled severe illness or medical conditions; concurrent or planned treatment with potent inhibitors or inducers of cytochrome P450 3A4/5; treatment with any investigational drug within 4 weeks prior to enrolment; of reproductive potential and not implementing an accepted and effective method of contraception | All 104 patients completed at least one AQoL questionnaire | Not reported | Not reported | A major limitation of the QoL data is that as the AQoL-8D questionnaire was administered to patients only whilst they remained on treatment, fewer patients contributed to data at cycle 10 than did at baseline due to withdrawal of patients from the study over the time. As a result, patients that tolerated treatment or did not progress were overrepresented with increasing number of treatment cycles. However, the results do suggest that for patients continuing on cabazitaxel treatment without disease progression, QoL was maintained and there was no evidence that the benefits of cabazitaxel may be outweighed by adverse effects on QoL |
| 502-Bahl-2015 | mCRPC patients: n= 112 | Patients were recruited from 12 sites in the UK. Patients were included if they had confirmed mCRPC previously treated with a docetaxel-containing regimen and experienced disease progression before or after docetaxel | Patients were included if they had confirmed mCRPC previously treated with a docetaxel-containing regimen and had experienced disease progression during or after docetaxel. Other inclusion criteria were ongoing surgical or medical castration; Eastern Cooperative Oncology Group (ECOG) performance status 0, 1 or 2; life expectancy ≥3 months; adequate bone marrow, renal and liver function; and written, informed consent. The exclusion criteria matched those of the TROPIC trial | 106 patients reported at least baseline EQ-5D questionnaire | Not reported | Not reported | It is acknowledged that the number of patients assessed at each point decreases with cycle number, indicating those patients who had discontinued treatment earlier than the planned 10 cycles. Like the other Australian EAP, as a result, patients that tolerated treatment or did not progress were overrepresented with increasing number of treatment cycles |
| 9U-Saad-2022 | mCRPC patients assessed for eligibility: n= 171  enrolled: n = 142  Olaparib arm: n = 71  Placebo + abiraterone arm: n = 71 | mCRPC patients after docetaxel and up to one additional line of previous chemo were recruited from 41 urological sites across 11 countries in North America and Europe | Eligible patients were aged 18 years or older with histologically or cytologically proven metastatic castrationresistant prostate cancer (defined as increasing prostatespecific antigen [PSA] concentration or other signs of disease progression despite androgen-deprivation therapy and serum testosterone concentrations at castrate levels [≤50 ng/dL], and with at least one metastatic lesion on bone scan, CT, or MRI), and who had received previous treatment with docetaxel and up to one additional line of previous chemotherapy. Eligible patients had an Eastern Cooperative Oncology Group performance status of 0–2 with no observed deterioration in the 2 weeks before the study and a life expectancy of at least 12 weeks. Patients were excluded if they had previously received more than two lines of chemotherapy or exposure to secondgeneration antihormonal drugs or olaparib | 100% of patients in both Olaparib and placebo group responded to EQ-5D-5L questionnaire at baseline. By end of study (Week 96), only 5 patients in the Olaparib and 4 in the placebo group reported a EQ-5D VAS score | Olaparib arm: 2 lost to follow-up  Placebo arm: 1 lost to follow-up | Not reported for EQ-5D. For FACT-P, If less than half of the subscale items were missing from a returned questionnaire, the subscale score was calculated by replacing the missing items with the mean of the non-missing items in the scale. If 50% or more of the items were missing, that visit was treated as missing | A limitation of the study is that all PRO analyses were exploratory, and the study was not powered for pain and HRQOL endpoints. In addition, patient numbers were low, with some imbalances in patient characteristics between the study groups, which could limit the generalisability of our findings. Additionally, intercurrent events, such as death and comorbidities, were not taken into account in the analysis, and these events could have affected the time to deterioration, since deterioration in HRQOL could be due to comorbidities. In addition, EQ-5D was not well reported in the paper or in the supplementary data. Methods surrounding this questionnaire use was limited |
| 10U-Saad-2022 | mCRPC patients reeiving talazoparib: n= 97 | Men with mCRPC harboring DDR gene alterations involved either directly or indirectly in HRR | Briefly, men with measurable soft-tissue disease were enrolled if they met the following criteria: (1) had mCRPC and harbored gene alterations in one or more of 11 DDR/HRR genes (ATM, ATR, BRCA1, BRCA2, CHEK2, FANCA, MLH1, MRE11A, NBN, PALB2, or RAD51C) likely to sensitize to PARPi; (2) were previously treated with one to two taxane-based chemotherapy regimens for advanced prostate cancer; and (3) progressed on one or more novel hormonal therapies in mCRPC | 100% response for EQ-5D-5L questionnaire (at least one) | Not reported | Not reported | This study is limited by its small sample size, the heterogeneity of the different disease molecular subtypes, and the lack of a control arm, making it challenging to differentiate regression to the mean from a benefit from talazoparib (and from other parallel antisymptom interventions). However, phase 3 studies of talazoparib plus novel hormonal therapy with a comparator arm are ongoing |

Abbreviations: AA = abiraterone acetate; AE = adverse event; BPI-SF = Brief Pain Inventory (Short Form); EORTC QLQ-C30 = European Organization for Research and Treatment of Cancer Quality of Life of Cancer Patients Questionnaire; EORTC -QLQ-PR25 = European Organisation for Research and Treatment of Cancer Quality of Life Questionnaire - Prostate Cancer Module; EQ-5D = EuroQoL 5-Dimensons; EQ-5D-3L = EuroQoL 5-Dimensions 3-Level; EQ-5D-5L = EuroQoL 5-Dimensions 5-Level; FACT-G = Functional Assessment of Cancer Therapy – General; FACT-P = Functional Assessment of Cancer Therapy – Prostate Cancer; HRQoL – Health-related quality of life; ITT = intent to treat; mCRPC – metastatic castration-resistant prostate cancer; NA = not applicable; NR = not reported; PCS = prostate cancer subscale; PROs = patient reported outcomes; QoL = quality of life; UK = United Kingdom;VAS = visual analog scale

Table S7: Quality Assessment – Economic Evaluations

|  | 34-Okamura-2021 | 421-Hill-2019 | 867-Holko-2014 | 869-Gong-2014 |
| --- | --- | --- | --- | --- |
| Study Design | | | | |
| The research question is stated | Y | Y | Y | Y |
| The economic importance of the research question is stated | Y | Y | Y | Y |
| The viewpoint(s) of the analysis are clearly stated and justified | Y | Y | Y | Y |
| The rationale for choosing the alternative programmes or interventions compared is stated | Y | Y | Y | Y |
| The alternatives being compared are clearly described? | Y | Y | N | N |
| The form of economic evaluation used is stated | Y | Y | Y | Y |
| The choice of form of economic evaluation is justified in relation to the questions addressed | Y | Y | Y | Y |
| Data Collection | | | | |
| The source(s) of effectiveness estimates used are stated | Y | Y | Y | Y |
| Details of the design and results of effectiveness study are given (if based on a single study) | Not appropriate | Not appropriate | Y | Not appropriate |
| Details of the methods of synthesis or meta-analysis of estimates are given (if based on an overview of a number of effectiveness studies) | N | N | Not appropriate | N |
| The primary outcome measure(s) for the economic evaluation are clearly stated | Y | N | Y | Y |
| Methods to value benefits are stated | Y | Y | Y | Y |
| Details of the subjects from whom valuations were obtained are given? | Y | Y | Y | Y |
| Productivity changes (if included) are reported separately | Not appropriate | Not appropriate | Not appropriate | N |
| The relevance of productivity changes to the study question is discussed | Not appropriate | Not appropriate | Not appropriate | N |
| Quantities of resources are reported separately from their unit costs | Y | N | Y | Y |
| Methods for the estimation of quantities and unit costs are described? | Y | Y | Y | Y |
| Currency and price data are recorded | Y | Y | Y | Y |
| Details of currency of price adjustments for inflation or currency conversion are given | N | Y | Y | Y |
| Details of any model used are given | Y | Y | Y | Y |
| The choice of model used and the key parameters on which it is based are justified | Y | Y | Y | Y |
| Analysis and Interpretation of Results *(Y/N/Not clear/Not appropriate)* | | | | |
| Time horizon of costs and benefits is stated | Y | N | Y | Y |
| The discount rate(s) is stated | Y | N | Y | Y |
| The choice of discount rate(s) is justified | Y | N | Y | Y |
| An explanation is given if costs and benefits are not discounted | Not appropriate | N | Not appropriate | Not appropriate |
| Details of statistical tests and confidence intervals are given for stochastic data | N | N | Y | N |
| The approach to sensitivity analysis is given | Y | N | Y | Y |
| The choice of variables for sensitivity analysis is justified | N | N | N | N |
| The ranges over which the variables are varied are justified | N | N | N | Y |
| Relevant alternatives are compared | Y | Y | Y | Y |
| Incremental analysis is reported | Y | N | Y | N |
| Major outcomes are presented in a disaggregated as well as aggregated form | N | N | N | N |
| The answer to the study question is given | Y | Y | Y | Y |
| Conclusions follow from the data reported | Y | Y | Y | Y |
| Conclusions are accompanied by the appropriate caveats | Y | Y | Y | Y |

|  | 2-Zhang-2021 | 8-ten Ham-2021 | 70-Su-2020 | 210-Barqawi-2019 |
| --- | --- | --- | --- | --- |
| Study Design | | | | |
| The research question is stated | Y | Y | Y | Y |
| The economic importance of the research question is stated | Y | Y | Y | Y |
| The viewpoint(s) of the analysis are clearly stated and justified | Y | Y | Y | Y |
| The rationale for choosing the alternative programmes or interventions compared is stated | Y | Y | Y | Y |
| The alternatives being compared are clearly described? | Y | Y | N | Y |
| The form of economic evaluation used is stated | Y | Y | Y | Y |
| The choice of form of economic evaluation is justified in relation to the questions addressed | Y | Y | Y | Y |
| Data Collection | | | | |
| The source(s) of effectiveness estimates used are stated | Y | Y | Y | Y |
| Details of the design and results of effectiveness study are given (if based on a single study) | N | N | N | Y |
| Details of the methods of synthesis or meta-analysis of estimates are given (if based on an overview of a number of effectiveness studies) | Not Appropriate | Not Appropriate | Not appropriate | Not Appropriate |
| The primary outcome measure(s) for the economic evaluation are clearly stated | Y | Y | Y | Y |
| Methods to value benefits are stated | Y | Y | Y | Y |
| Details of the subjects from whom valuations were obtained are given? | Y | Y | Not Clear | Y |
| Productivity changes (if included) are reported separately | Not appropriate | Not appropriate | Not appropriate | Not Appropriate |
| The relevance of productivity changes to the study question is discussed | Not appropriate | Not appropriate | Not appropriate | Not Appropriate |
| Quantities of resources are reported separately from their unit costs | N | N | N | N |
| Methods for the estimation of quantities and unit costs are described? | Y | Y | Y | Y |
| Currency and price data are recorded | Y | Y | Y | Y |
| Details of currency of price adjustments for inflation or currency conversion are given | N | Y | Y | Y |
| Details of any model used are given | Y | Y | Y | Y |
| The choice of model used and the key parameters on which it is based are justified | Y | Y | Y | Y |
| Analysis and Interpretation of Results *(Y/N/Not clear/Not appropriate)* | | | | |
| Time horizon of costs and benefits is stated | Y | Y | Y | Y |
| The discount rate(s) is stated | Y | Y | Y | Y |
| The choice of discount rate(s) is justified | N | Y | Y | Y |
| An explanation is given if costs and benefits are not discounted | Not appropriate | Not Appropriate | Not appropriate | Not appropriate |
| Details of statistical tests and confidence intervals are given for stochastic data | N | Y | Y | Y |
| The approach to sensitivity analysis is given | Y | Y | Y | Y |
| The choice of variables for sensitivity analysis is justified | N | Y | Y | Y |
| The ranges over which the variables are varied are justified | N | N | Y | Y |
| Relevant alternatives are compared | Y | Y | Y | Y |
| Incremental analysis is reported | Y | Y | Y | Y |
| Major outcomes are presented in a disaggregated as well as aggregated form | Y | N | N | N |
| The answer to the study question is given | Y | Y | Y | Y |
| Conclusions follow from the data reported | Y | Y | Y | Y |
| Conclusions are accompanied by the appropriate caveats | Y | Y | Y | Y |

|  | 396-Sanyal-2016 | 511-Wilson-2014 | 573-Zhong-2013 | 46U-Li-2021 |
| --- | --- | --- | --- | --- |
| Study Design | | | | |
| The research question is stated | Y | Y | Y | Y |
| The economic importance of the research question is stated | Y | Y | Y | Y |
| The viewpoint(s) of the analysis are clearly stated and justified | Y | Y | Y | Y |
| The rationale for choosing the alternative programmes or interventions compared is stated | N | Y | Y | Y |
| The alternatives being compared are clearly described? | Y | Y | Y | Y |
| The form of economic evaluation used is stated | Y | Y | Y | Y |
| The choice of form of economic evaluation is justified in relation to the questions addressed | Y | Y | Y | Y |
| Data Collection | | | | |
| The source(s) of effectiveness estimates used are stated | N | Y | Y | Y |
| Details of the design and results of effectiveness study are given (if based on a single study) | N | Y | Y | Y |
| Details of the methods of synthesis or meta-analysis of estimates are given (if based on an overview of a number of effectiveness studies) | N | Not Appropriate | Not appropriate | Not Appropriate |
| The primary outcome measure(s) for the economic evaluation are clearly stated | Y | Y | Y | Y |
| Methods to value benefits are stated | Y | Y | Y | Y |
| Details of the subjects from whom valuations were obtained are given? | Y | Y | Y | Y |
| Productivity changes (if included) are reported separately | Not appropriate | Not appropriate | Not appropriate | Not Appropriate |
| The relevance of productivity changes to the study question is discussed | Not appropriate | Not appropriate | Not appropriate | Not Appropriate |
| Quantities of resources are reported separately from their unit costs | N | Y | N | N |
| Methods for the estimation of quantities and unit costs are described? | Y | Y | Y | Y |
| Currency and price data are recorded | Y | Y | Y | Y |
| Details of currency of price adjustments for inflation or currency conversion are given | Y | Y | Y | Y |
| Details of any model used are given | Y | Y | Y | Y |
| The choice of model used and the key parameters on which it is based are justified | Y | Y | Y | Y |
| Analysis and Interpretation of Results *(Y/N/Not clear/Not appropriate)* | | | | |
| Time horizon of costs and benefits is stated | Y | Y | Y | Y |
| The discount rate(s) is stated | Y | Y | Y | Y |
| The choice of discount rate(s) is justified | N | Y | Y | N |
| An explanation is given if costs and benefits are not discounted | Not appropriate | Not Appropriate | Y | Not appropriate |
| Details of statistical tests and confidence intervals are given for stochastic data | Y | N | N | Y |
| The approach to sensitivity analysis is given | Y | Y | Y | Y |
| The choice of variables for sensitivity analysis is justified | N | Y | Y | Y |
| The ranges over which the variables are varied are justified | N | N | Y | Y |
| Relevant alternatives are compared | Y | Y | Y | Y |
| Incremental analysis is reported | Y | Y | Y | Y |
| Major outcomes are presented in a disaggregated as well as aggregated form | Y | N | N | N |
| The answer to the study question is given | Y | Y | Y | Y |
| Conclusions follow from the data reported | Y | Y | Y | Y |
| Conclusions are accompanied by the appropriate caveats | Y | Y | Y | Y |

|  | 396-Sanyal-2016 |
| --- | --- |
| Study Design | |
| The research question is stated | Y |
| The economic importance of the research question is stated | Y |
| The viewpoint(s) of the analysis are clearly stated and justified | Y |
| The rationale for choosing the alternative programmes or interventions compared is stated | N |
| The alternatives being compared are clearly described? | Y |
| The form of economic evaluation used is stated | Y |
| The choice of form of economic evaluation is justified in relation to the questions addressed | Y |
| Data Collection | |
| The source(s) of effectiveness estimates used are stated | Y |
| Details of the design and results of effectiveness study are given (if based on a single study) | Y |
| Details of the methods of synthesis or meta-analysis of estimates are given (if based on an overview of a number of effectiveness studies) | Not Appropriate |
| The primary outcome measure(s) for the economic evaluation are clearly stated | Y |
| Methods to value benefits are stated | Y |
| Details of the subjects from whom valuations were obtained are given? | Y |
| Productivity changes (if included) are reported separately | Not appropriate |
| The relevance of productivity changes to the study question is discussed | Not appropriate |
| Quantities of resources are reported separately from their unit costs | N |
| Methods for the estimation of quantities and unit costs are described? | Y |
| Currency and price data are recorded | Y |
| Details of currency of price adjustments for inflation or currency conversion are given | Y |
| Details of any model used are given | Y |
| The choice of model used and the key parameters on which it is based are justified | Y |
| Analysis and Interpretation of Results *(Y/N/Not clear/Not appropriate)* | |
| Time horizon of costs and benefits is stated | Y |
| The discount rate(s) is stated | Y |
| The choice of discount rate(s) is justified | Y |
| An explanation is given if costs and benefits are not discounted | Not appropriate |
| Details of statistical tests and confidence intervals are given for stochastic data | N |
| The approach to sensitivity analysis is given | Y |
| The choice of variables for sensitivity analysis is justified | N |
| The ranges over which the variables are varied are justified | N |
| Relevant alternatives are compared | Y |
| Incremental analysis is reported | Y |
| Major outcomes are presented in a disaggregated as well as aggregated form | Y |
| The answer to the study question is given | Y |
| Conclusions follow from the data reported | Y |
| Conclusions are accompanied by the appropriate caveats | Y |

Table S8: Utility Outcomes in all Primary Studies

| **Region** | **Trial; NCT; Reference** | **Study Type** | **Patient Population** | **LoT** | **Treatment Arm** | **Timepoints** | **Outcome** | **Baseline^A^** | **Utility value at last timepoint^A^** | **Change from baseline*** |
| --- | --- | --- | --- | --- | --- | --- | --- | --- | --- | --- |
| China, Hong Kong, Republic of Korea, Taiwan | NCT02294461  Pu, 2022 | Phase 3 RCT | Asymptomatic or mildly symptomatic metastatic prostate cancer and disease progression despite ADT | 1L | Enzalutamide (n=198) | Baseline, week 13, 25, 37, and 49 | EQ VAS | 74.8 (16) | 76.5 (17.7) | NR |
|  |  |  |  |  | Placebo (n=190) |  |  | 76.6 (15.9) | 71.2 (28.7) | NR |
| Global | TERRAIN  NCT01288911  Heidenreich, 2017 | Phase 2 RCT | Chemotherapy-naïve mCRPC | 1L | Enzalutamide 160mg (n=184) | EQ-5D was collected baseline; follow up scores measured up to 61 weeks. | EQ-5D-5L  EQ VAS | EQ-5D-5L: 0.81 (0.2)  EQ VAS: 77.7 (15.48) | NR | EQ-5D-5L^B^: -0.11 (0.03)  EQ-5D-5L^C^: -0.19 (0.04)  EQ VAS^B^:  -3.24 (1.56)  EQ VAS^C^:  -8.78 (2.12) |
|  |  |  |  |  | Bicalutamide 50mg (n=191) |  |  | EQ-5D-5L: 0.83 (0.18)  EQ VAS: 76.9 (17.73) | NR | EQ-5D-5L^B^: -0.10 (0.04)  EQ-5D-5L^C^: -0.24 (0.05)  EQ VAS^B^:  -2.62 (2.27)  EQ VAS^C^:  -11.25 (2.95) |
| Global | PREVAIL  NCT01212991  Loriot, 2015  Devlin, 2017 | Phase 3 RCT | Chemotherapy-naïve, asymptomatic/ minimally symptomatic mCRPC | 1L | Enzalutamide 160mg (n=872) | EQ-5D was collected at baseline and at regular intervals throughout the trial until week 61 | EQ-5D (unspecified)  EQ VAS | EQ-5D:  0.85 (0.15)  EQ VAS: 77.15 (16.72) | NR | EQ-5D^D^:  -0.10 (-0.09 to -0.05)  EQ VAS^D^:  -5.19 (-7.14 to -3.23) |
|  |  |  |  |  | Placebo (n=845) |  |  | EQ-5D:  0.84 (0.17)  EQ VAS:  75.94 (17.53) | NR | EQ-5D^D^:  -0.10 (-0.14 to -0.06)  EQ VAS^D^:  -9.76 (-12.61 to -6.92) |
| Global | AFFIRM or PREVAIL:  NCT01212991  Saad, 2017 | SRE Population Subgroup Analysis | Chemotherapy-naïve, asymptomatic/ minimally symptomatic mCRPC with skeletal-related events (SRE) | !L | Enzalutamide (n=1008) | NR | EQ-5D | EQ-5D: 0.829 (0.154) | NR | Any SRE:  -0.11 |
| UK | Lloyd, 2015 | Cross-sectional Survey | Asymptomatic or mildly symptomatic mCRPC | 1L | Chemotherapy (docetaxel or other) (n=163) | Before chemotherapy, during chemotherapy^E^, after chemotherapy^E^ | EQ-5D-5L  EQ VAS | Before chemo:  EQ-5D-5L:  0.830 (0.126)  EQ VAS:  77.5 (12.6) | During Chemo:  EQ-5D-5L:  0.692 (0.219)  EQ VAS:  67.4 (14.3)  After Chemo:  EQ-5D-5L:  0.700 (0.183)  EQ VAS:  66.0 (17.9) | NR |
| Europe | Diels, 2015 | Cross-sectional observational study | Chemotherapy-naïve mCRPC | 1L | Unspecified treatment | Chemo-naive, during chemotherapy, after chemotherapy | EQ-5D-3L (SE) | Chemo-naïve:  EQ-5D-3L:  0.70 (0.02)  All patients:  EQ-5D-3L:  0.66 (0.01) | During chemo:  EQ-5D-3L:  0.66 (0.02)  After chemo:  EQ-5D-3L:  0.6 (0.03) | NR^F^ |
| Europe | PREMISE:  NCT02495974  Payne, 2021 | Prospective observational study | Patients with mCRPC who were prescribed enzalutamide as a part of routine clinical practice | 1L, 2L or 3L | 1L Enzalutamide (chemo-naïve + abiraterone naïve) (n=1109) | 3, 6, and 9 months | EQ-5D-5L  EQ VAS | EQ-5D-5L:  0.71 (0.24)  EQ VAS:  68.0 (19.0) | EQ-5D-5L:  0.76 (0.20)  EQ VAS:  71.9 (17.5) | NR |
|  |  |  |  |  | 2L Enzalutamide post-chemo (n=398) |  |  | EQ-5D-5L: 0.68 (0.24)  EQ VAS: 66.2 (20.4) | EQ-5D-5L: 0.73 (0.23)  EQ VAS: 71.8 (19.5) |  |
|  |  |  |  |  | 2L Enzalutamide post-abiraterone (n=42) |  |  | EQ-5D-5L: 0.67 (0.27)  EQ VAS: 63.6 (20.8) | EQ-5D-5L: 0.83 (0.13)  EQ VAS: 65.0 (18.6) |  |
|  |  |  |  |  | 3L Enzalutamide post chemo and abiraterone (n=94) |  |  | EQ-5D-5L: 0.63 (0.24)  EQ VAS: 62.7 (20.1) | EQ-5D-5L: 0.65 (0.32)  EQ VAS: 61.0 (25.0) |  |
| UK, France, Germany | Dearden, 2019 | Cross-sectional survey with semi-structured interviews | Pre-chemotherapy setting for mCRPC | 1L | Enzalutamide (n=49) | NR | EQ-5D-5L  EQ VAS | EQ-5D-5L:  0.79 (NR)  EQ VAS:  68.6 (NR) | NR^F^ | NR^F^ |
|  |  |  |  |  | Abiraterone acetate + prednisone (n=38) |  |  | EQ-5D-5L:  0.87 (NR)  EQ VAS:  72.8 (NR) | NR^F^ |  |
| Italy | ABItude  Procopio, 2020  Procopio, 2022 | Prospective observational study | Asymptomatic/mildly symptomatic, chemotherapy-naïve mCRPC | 1L | Abiraterone acetate + prednisone (n=481) | Baseline, 24 months | EQ-5D-3L  EQ VAS | EQ-5D-3L median (IQR): 0.9 (0.8-1.0)  EQ VAS median (IQR): 70.0 (50.0-80.0) | EQ-5D-3L median (IQR): 0.9 (0.8-1.0)  EQ VAS median (IQR): 75 (60-80) | NR |
| UK | Early Access Program (EAP)  NCT01254279  Bahl, 2015 | Single-arm trial | mCRPC patients with disease progression during or after docetaxel | 2L | Cabazitaxek + prednisone (n=112) | Baseline, Cycles 2-8, end of therapy | EQ-5D-3L | EQ-5D-3L:  0.699 (0.044) | EQ-5D-3L:  0.819 (0.075) | NR |
| Global | CARD:  NCT02485691  Kramer, 2020  Fizazi, 2020 | Randomized, open label trial | Patients with mCRPC who had progressed on docetaxel and within 12 months on a prior alternative androgen-signaling targeted inhibitor (ARTA) | 3L | Cabazitaxel (n=129) | Baseline, Cycles 2-8, end of therapy | EQ-5D-5L  EQ VAS | EQ-5D-5L:  0.7 (0.26)  EQ VAS:  65.8 (20.4) | NR | -0.049 (-0.166, 0.010) |
|  |  |  |  |  | Abiraterone or enzalutamide (n=126) |  |  | EQ-5D-5L:  0.7 (0.22)  EQ VAS:  66.3 (18.5) | NR | -0.110 (-0.100, 0.063) |
| Netherlands | PRO-CAPRI:  NL3934  Kuppen, 2020 | Observational, prospective | Patients diagnosed with mCRPC between January 1, 2010 and December 31, 2016 | 1L+ | All patients (n=167) | Month 3, 6, 9, 12, 15, 18, 21, 24 | EQ-5D  EQ VAS | EQ-5D: 0.82 (0.17)  EQ VAS: 73.2 (17) | NR | NR |
|  |  |  |  |  | Chemo-naïve^G^ (n=112) |  |  | EQ-5D: 0.82 (0.16)  EQ VAS: 72.9 (17) |  | NR |
|  |  |  |  |  | Post-chemo^H^ (n=39) |  |  | EQ-5D: 0.82 (0.16)  EQ VAS: 73.9 (16) |  | NR |
| Australia | Australian EAP:  NCT01254279  Parente, 2017 | Observational | Patients previously treated with a docetaxel-containing regimen receiving cabazitaxel through the Australian EAP | 2L+ | Cabazitaxel (n=104) | Baseline, End of therapy | AQoL | Mean baseline: 0.7 | Mean EoT: 0.68 | Mean change (SD): -0.02 (0.15) |
| Global | Skaltsa, 2014  Questionnaire from AFFIRM trial | Utility mapping | Patients with mCRPC who received prior chemotherapy | 2L+ | Unspecified (n=209) | NR | EQ-5D | EQ-5D (mean): 0.688 (0.282)  EQ-5D (median): 0.727 | NR | NR |
| Global | Wu, 2007 | Utility Mapping | Patients with metastatic hormone-refractory prostate cancer | Unknown | Unspecified (n=NR) | Baseline only | QLQ-C30 to EQ-5D | 0.635 (0.309) | NR | NR |
| Japan | Murasawa, 2019 | Cross-sectional observational | Patients with local PC, localized progressive PC, distant metastatic PC, and distant mCRPC (only mCRPC extracted) | Unknown | Unspecified (n=38) | Baseline | EQ-5D-5L  EQ VAS | EQ-5D-5L:  0.84 (0.17)^F^  EQ VAS:  66.3 (20.4) | NR | NR |
| Global | NCT01972217  Saad, 2022 | Phase 2 Trial | mCRPC patients after docetaxel and up to one additional line of previous chemo | 3L | Olaparib, abiraterone, and prednisone/prednisolone (n=71) | NA | EQ VAS | EQ VAS:  65.0 (20.9) | NR | NR |
|  |  |  |  |  | Placebo, abiraterone, and prednisone/prednisolone (n=71) |  |  | EQ VAS:  68.0 (16.5) |  |  |
| Global | TALAPRO-1  NCT03148795  Saad, 2022 | Single-arm trial | Men with mCRPC harboring DDR gene alterations involved either directly or indirectly in HRR | 2L or 3L | All patients – Talazoparib (n=97) | Baseline to disease progression | EQ-5D-5L  EQ VAS | NR | NR | EQ-5D-5L:  0.05 (0.01, 0.08)  EQ VAS:  5.42 (2.65, 8.18) |
|  |  |  |  |  | BRCA1/2 - Talazoparib (n=56) |  |  | NR | NR | EQ-5D-5L:  0.07 (0.03, 0.1)  EQ VAS:  4.74 (1.07, 8.41) |
| Global | Boye, 2022 | Real-world Study | Patients with mCRPC or mHSPC (only mCRPC extracted) | Unspecified | Unspecified (n=331) | Baseline | EQ-5D-5L  EQ VAS | EQ-5D-5L:  0.72  EQ VAS:  53.4 | NR | NR |
| South Korea | Kim, 2022 | Utility Weights Estimation | Patients with prostate-related conditions in Korea  Patients in metastatic castration-resistant prostate cancer health state | Unknown | Unspecified (n=456) | NR | Standard Gamble | Standard Gamble, mean (SD)/median:  0.281±0.269/0.200  VAS, mean (SD)/median:  0.110±0.184/0.69 | NR | NR |

^a^ Reported as mean (SD) unless otherwise specified

^b^ Mixed model for repeated measures least square mean change from baseline (SE)

^c^ Pattern mixture model least square mean change from baseline (SE)

^d^ Mixed-effects model for repeated measures adjusted mean change from baseline (95% CI)

^e^ Cross-sectional study enrolled patients at differing stages of the first-line treatment pathway. Values at “follow-up” (During and after chemotherapy) do not represent follow-up values for patients providing baseline utility values and vice versa

^f^ Due to the cross-sectional native of the study, data were measured at only one timepoint (baseline)

^g^ The mCRPC chemotherapy naïve state was defined as metastatic disease before chemotherapy treatment, usual treatment, symptoms (pain and fatigue), side effects, and concern about continued cancer spread.

^h^ The mCRPC post-chemotherapy state was defined as patients currently receiving chemotherapy or post chemotherapy

Abbreviations: ADT = androgen deprivation therapy; CI = confidence interval; DCE = discrete choice experiments; EOT = end of therapy; EQ-5D = EuroQuol 5-Dimension; EQ-5D-3L = EuroQuol 5-Dimension 3-Level; EQ-5D-5L = EuroQuol 5-Dimension 5-Level; HRQoL= health-related quality of life; IQR = interquartile range; mCRPC = metastatic castration-resistant prostate cancer; NA = not applicable; NR = not reported; NS = non-significant; PRO = patient reported outcome; RCT = randomized control trial; SD = standard deviation; SE = standard error; TTO = time trade-off; UK = United Kingdom; US = United States; VAS = visual analogue scale.

Table S9: Utility Outcomes in Economic Evaluations

| **Region** | **Reference** | **Study Type** | **Patient Population** | **Treatment Arm** | **Source of Utilities** | **Reported Utility Values** |
| --- | --- | --- | --- | --- | --- | --- |
| US | Gong, 2014 | CUA | Asymptomatic/mildly symptomatic, pre-docetaxel mCRPC | Abiraterone acetate | Societal, non-patient responses to QoL questionnaires related to mCRPC  Bremner et al. (2007) | Stable disease**^a^**: 0.76  Progressed disease**^b^**: 0.65 Death: 0  Disutility due to AEs: -0.008 |
|  |  |  |  | Sipuleucel-T |  | Stable disease**^a^**: 0.76  Progressed disease**^b^**: 0.65 Death: 0  Disutility due to AEs: -0.008 |
|  |  |  |  | Prednisone |  | Stable disease**^a^**: 0.76  Progressed disease**^b^**: 0.58 Death: 0  Disutility due to AEs: -0.004 |
| Canada | Hill, 2019 | CUA | First-line mCRPC | Enzalutamide 160mg/day  Metformin 1000mg/day | Lloyd et al. (2015)  Diels et al. (2015)  Vincente et al. (2015)  Davies et al. (2015) | **Treatment Utility** First-line treatment for mCRPC: 0.83  **Treatment-Induced Complications**  Cerebrovascular accident disutility: -0.15  **Second-line Treatment Utility**  Second-line treatment pre-chemotherapy: 0.70  Chemotherapy: 0.66  Post-chemotherapy: 0.6  BSC: 0.5 |
| Japan | Okumura, 2021 | CUA | Japanese patients with chemotherapy-naïve mCRPC | Enzalutamide | Literature review results reported by Wolff et al., 2015 Sandblom et al., 2004 Swinnburn et al., 2010 PREVAIL (NCT01212991) trial | QoL Gain (both enzalutamide and abiraterone^c^): 0.022 QoL reduction at adverse event: -0.153 to -0.069 Stable disease: 0.844 Post-progression 1^d^: 0.64 Post-progression 2^d^: 0.66 Palliative therapy: 0.5 QoL reduction at SRE: -0.237 to -0.056 |
|  |  |  |  | Abiraterone acetate + prednisone |  |  |
|  |  |  |  | Docetaxel + prednisone |  |  |
|  |  |  |  | Mitoxantrone + prednisone |  |  |
| US | Zhang, 2021 | CUA | Patients with mCRPC previously treated with docetaxel who had progression within 12 months while receiving an alternative inhibitor (abiraterone or enzalutamide) | Cabazitaxel | Utilities:  Barqawi et al. 2019  Efficacy Data: CARD trial | Progressive disease on 2L therapy: 0.37  2L Treatment: 0.617^e^ |
|  |  |  |  | Abiraterone acetate or Enzalutamide |  |  |
| Nethelands | Ten Ham, 2021 | CUA | mCRPC treated with abiraterone acetate in the Netherlands | 1L+ Abiraterone acetate | Utilities:  van der Graaff M. Farmacotherapeutic Report Abirateron (Zytiga) bij gemetastaseerd  castratieresistent prostaatcarcinoom mCRPC [in Dutch]. Vol. 1.  Diemen, The Netherlands: 2011. | PFS Utility: 0.84  Progressed Disease Utility: 0.715  Disutility (First Cycle): 0.052  Disutility (> First Cycle): 0.047 |
|  |  |  |  | 2L+ Docetaxel |  | Disutlity (First Cycle): 0.043  Disutility (> First Cycle): 0.016 |
|  |  |  |  | 2L+ Cabazitaxel |  | Disutilty (First Cycle): 0.185  Disutlity (> First Cycle): 0.031 |
|  |  |  |  | 2L+ Enzalutamide |  | Disutility (First Cycle): 0.016  Disutility (> First Cycle): 0.015 |
|  |  |  |  | 2L+ Radium-223 |  | Disutility (First Cycle): 0.052  Disutility (> First Cycle): 0.037 |
| US | Ko, 2021 | CUA | mCRPC patients with at least one homologous recombination repair (HRR) gene alteration and who have experience progression after novel hormone therapy | Olaparib, abiraterone acetate, and enzalutamide | Efficacy: PROfound trial  Utilities:  De Bono et al. (2020)  Lloyd et al. (2015)  Swinburn et al. (2010)  Wehler et al. (2018)  Hall et al. (2019) | Pre-progression: 0.83^f^  Post-progression: 0.63^g^  Death: 0.0 |
| US | Su, 2020 | CUA | mCRPC patients with at least one homologous recombination repair (HRR) gene alteration and who have experienced disease progression after novel hormonal therapy | Olaparib, abiraterone acetate, and enzalutamide | Efficacy: PROfound trial  Utilities:  Bremner et al. (2007)  Barqawi et al. (2019)  Amdahl et al. (2016) | Progression-free disease (mCRPC): 0.76  Progressed disease for mCRPC: 0.37  Disutlity (Grade ½ AEs): 0.01  Disutility (>=Grade 3 AEs): 0.16 |
| US | Barqawi, 2019 | CUA | Patients with visceral metastatic CRPC post-docetaxel therapy resistance | Abiraterone acetate and prednisone  Cabazitaxel and prednisone  Enzalutamide | Efficacy:  COU-AA-301  TROPIC  AFFIRM  Utilities:  Peters et al. (2018)  Gharaibeh et al. (2017)  Swinburn et al. (2010) | PFS (2L): 0.617  Progressed disease on 2L: 0.37  Disutilities:  Anemia: -0.119  Diarrhea: -0.212  Fatigue: -0.473  Backpain: -0.067  Neutropenia: -0.131  Bone pain: -0.067 |
| Canada | Sanyal, 2016 | CUA | Men with localized and advanced prostate cancer | Unspecified | Pataky et al. (2014)  Yong (2012) | mCRPC health state: 0.5  Death or Pca: 0  Disutility of mCRPC treatment complications: -0.11 |
| US | Wilson, 2014 | CUA | mCRPC patients who failed previous docetaxel therapy | Cabazitaxel and prednisone  Enzalutamide (optional prednisone)  Abiraterone and prednisone  Placebo | Efficacy: TROPIC, AFFIRM, COU-AA-301  Utiltiies:  Konski (2009)  Dyer (2010)  Krahn (2003)  Nafees (2008) | Metastatic disease: 0.62^h^  Radiation (for pain): 0.67^h^  No pain: 0.69^h^  Disutilities^i^:  Bone pain: 0.43 (HUI)  Cardiac: 0.51 (EQ-5D)  Pain: 0.55 (QWB)  Neutropenia: 0.57 (SG)  Seizure: 0.58 (GOS)  No Radiation (for pain): 0.62 (QWB) |
| US | Holko, 2014 | CUA | Asymptomatic or minimally symptomatic castration-resistant | Sipuleucel-T with standard care | Efficacy: IMPACT  Utilities:  Bremner (2007) | Survival state utility: 0.76^j^ |
| US | Zhong, 2013 | CUA | Patients with mCRPC following docetaxel treatment failure | Abiraterone acetate  Cabazitaxel  Placebo (Prednisone)  Placebo (Mitoxantrone) | Efficacy: TROPIC, AFFIRM, COU-AA-301  Utiltiies:  Konski (2009)  Dyer (2010)  Krahn (2003)  Nafees (2008) | Metastatic disease: 0.62^h^  Radiation (for pain): 0.67^h^  No pain: 0.69^h^  Disutilities^i^:  Bone pain: 0.43 (HUI)  Cardiac: 0.51 (EQ-5D)  Pain: 0.55 (QWB)  Neutropenia: 0.57 (SG)  Seizure: 0.58 (GOS)  No Radiation (for pain): 0.62 (QWB) |
| US & China | Xu, 2022 | CUA | Subjects with mCRPC who had worsened conditions during mCRPC treatment and had previously received taxane-based chemotherapy | Olaparib  Control | HSUVs from Zhang 2021; Ramamurthy 2019; Hall 2019 | Disutility from AEs:  Anemia: -0.119;  Nausea: -0.21;  Fatigue: -0.09;  Vomiting: -0.21;  Back pain: -0.067;  UTI: -0.07  Progressive disease on 2L: 0.37  PFS on 2L: 0.617 |
| US | Li, 2021 | CUA | mCRPC patients with at least one gene mutation in BRCA1, BRCA2, or ATM and had progressed during enzalutamide or abiraterone acetate treatment | Olaparib  Control (Enzalutamide and abiraterone acetate) | Efficacy: PROfound  Utiltiies:  Cynthia (2014)  Barqawi et al (2019)  Doyle (2008)  Nafees (2008)  Welk (2018) | Progression-free survival on 2L: 0.76  Progressed disease on 2L: 0.65  Disutilities of AEs:  Anemia: -0.119;  Dyspnea: -0.05;  Vomiting: -0.092;  UTI: -0.016 |
| Canada | Sanyal, 2014 | Validation of economic evaluation model | Patients with PCa from diagnosis to end of life | Unspecified systemic therapy | Pataky, 2014 | mCRPC health state utility (LoT unknown): 0.85  End of life: 0.5 |

^a^ Stable disease is defined as lack of cancer progression

^b^ Progressed disease determined based on radiographic progression defined in the clinical trials

^c^ QoL gain utility value for abiraterone was assumed to be equal to enzalutamide, as stated by the authors

^d^ Post-progression 1 is defined as a patient who received a second line therapy, while post-progression 2 is defined as a patient who received a third line therapy.

^e^ PFS on 2L therapy

^f^ 2L pre-progression

^g^ 2L post-progression, moving to 3L

^h^ Disease in 2L, QWB values

^I^ Grade III/IV disutility due to side effect

^j^ According to source, this describes mCRPC patients with severe sexual symptoms

Abbreviations: AE = adverse event; BSC = best supportive care; CUA = cost-utility analysis; FACT-P= Functional Assessment of Cancer Therapy–Prostate; HRPC = hormone-refractory prostate cancer; mCRPC = metastatic castration-resistant prostate cancer; mHRPC = metastatic hormone-refractory prostate cancer; Q3W = once every three weeks; QoL = quality of life; SRE = skeletal-related event; UK = United Kingdom; US = United States; VAS = visual analog scale.

Table S10: Utility Outcomes in all HTA Submissions Containing an Economic Evaluation

| **Region** | **Reference** | **HTA Agency** | **Patient Population** | **Treatment Arm** | **Instrument** | **Source** | **Reported Utility Values** |
| --- | --- | --- | --- | --- | --- | --- | --- |
| England and Wales | G-HTA-485-NICE-2016  G-HTA-486-NICE-2016 | NICE | Asymptomatic/mildly symptomatic, chemotherapy naïve mCRPC | Abiraterone Acetate + Prednisone  Placebo (Prednisone) | FACT-P mapped to EQ-5D | UK mCRPC patient utility study (online survey)    Sandblom et al., 2004    COU-AA-302 | No or mild symptoms; chemotherapy not yet clinically indicated: 0.83  With symptoms, chemotherapy clinically indicated but not started: 0.63  Having chemotherapy: 0.69  After chemotherapy: 0.70  Utility gain for patients receiving abiraterone acetate: 0.021 |
| England and Wales | G-HTA-482-NICE-2015  G-HTA-483-NICE-2016 | NICE | Asymptomatic/mildly symptomatic, chemotherapy naïve mCRPC | Enzalutamide 160 mg  Placebo (BSC) | FACT-P^A^  EQ-5D^A^ | PREVAIL and published literature | Stable Disease  Enzalutamide: 0.866**^B^**  Placebo (BSC): 0.844  Post-progression 1**^C^**: 0.658  Post-progression 2**^D^**: 0.612  Palliative care: 0.5  Second-line Treatment Utility  Enzalutamide after docetaxel: 0.688 |
| England and Wales | Collins, 2007 | NICE | mHRPC (mCRPC) | Docetaxel (various doses and regimens) + Prednisone or Prednisolone  Mitoxantrone + Prednisone or Prednisolone | FACT-P to EQ-5D | Sandblom et al., 2004    NHS Value in Health Panel    Derived from FACT-P data | **Stable Disease^E^**  Value score in last year of life (mean): 0.538  VAS score (mean): 0.54 **Disutility values^F,G^** Docetaxel: 0.1347 Mitoxantrone: 0.0676 Estramustine: 0.1097  **Stages of HRPC^F^**  Early stage**^H,I^**: 0.725  Moderate stage**^H,I^**: 0.6159  Late stage**^H,I^**: 0.5774  Combined estimate: 0.638 |
| Scotland | G-HTA-629-SMC-2015 | SMC | Asymptomatic/mildly symptomatic, chemotherapy-naïve mCRPC | Abiraterone acetate (AA) + corticosteroid  Placebo/Watchful Waiting (WW) | EQ-5D | Utility values were based on a survey of 163 patients in the UK and used EQ-5D to collect quality of life scores to correspond to the various phases of the model (pre-docetaxel, on docetaxel, and post-docetaxel treatment). | Treatment Utility  Pre-docetaxel (AA or WW) (1L) 0.83  On docetaxel (2L): 0.692  Post-docetaxel treatment (Enzalutamide) (3L): 0.70  On-treatment utility gain for patients receiving abiraterone acetate plus prednisone in pre-docetaxel phase: 0.021 |
| UK | G-HTA-439-NICE-2020 | NICE | mCRPC patients who have received prior treatment with a taxane chemotherapy and NHA | Olaparib  Choice of NHA  Cabazitaxel | EQ-5D-3L | EQ-5D-5L data from the PROfound trial mapped to EQ-5D-3L utilities as recommended in the NICE reference case; SRE utility decrements from Fassler 2011 | Utility Decrements^J^:  Anaemia: 0.1250;  Infection: 0.0900;  Leukopenia: 0.0900;  Neutropenia: 0.0900;  Musculoskeletal pain or discomfort: 0.0690;  Thrombocytopenia: 0.0900;  Febrile neutropenia: 0.1200;  Diarrhoea: 0.0470;  Asthenia/fatigue: 0.0940;  spinal cord compression: 0.5550;  Pathological bone fracture: 0.1300;  Radiation to the bone: 0.0700;  Surgery to the bone: 0.1300  Other utilities were redacted |
| UK | G-HTA-468-NICE-2012  G-HTA-469-NICE-2012 | NICE | mCRPC with disease progression after one docetaxel-containing chemotherapy regimen  mCRPC previously treated with a docetaxel-containing regimen | Abiraterone acetate + prednisone  Mitoxantrone (+/- prednisone) | FACT-P mapped to EQ-5D | The manufacturer undertook a two-stage analysis to convert FACTP data from the COU-AA-301 trial into EQ-5D utility values | Post-progression on 2L+: 0.5  Pre-progression on 2L+: Confidential |
| UK | G-HTA-470-NICE-2014  G-HTA-471-NICE-2014 | NICE | mHRPC previously treated with a docetaxel-containing regimen | Enzalutamide  Abiraterone acetate + prednisone | FACT-P mapped to EQ-5D | Utility increase from FACT-P mapped to EQ-5D from AFFIRM; utility decrease from Sandblom 2004 | Utility decrease: -0.085^k^  Utility increase: 0.04^L^  Utility increase: confidential^M^ |
| UK | G-HTA-488-NICE-2016  G-HTA-489-NICE-2016 | NICE | Patients with mCRPC whose disease had progressed on or after treatment with docetaxel | Cabazitaxel | EQ-5D | HSUVs from UK early access program | Progressive disease on 2L: 0.63  Stable disease 2L First cycle: 0.7  Stable disease 2L Tenth cycle: 0.82  Last 3 months of life: 0 |
|  |  |  |  | Mitoxantrone |  |  | NR |
| Scotland | G-HTA-620-SMC-2016 | SMC | Adult patients with hormone refractory metastatic prostate cancer previously treated with a docetaxel-containing regimen | Cabazitaxel | EQ-5D | HSUVs from UK early access program | Progressive disease on 2L: 0.63  Stable disease 2L First cycle: 0.7  Stable disease 2L Tenth cycle: 0.82 |
|  |  |  |  | Mitoxantrone |  |  |  |
| Scotland | G-HTA-633-SMC-2013 | SMC | Adult men with mCRPC whose disease has progressed on or after docetaxel therapy | Enzalutamide  Abiraterone acetate | EQ-5D | Derived from EQ-5D data collected within the AFFIRM trial | Disutility of progressive disease: -0.085 |
| Scotland | G-HTA-667-SMC-2022 | SMC | Previously treated BRCA mutation-positive hormone-relapsed metastatic prostate cancer | Olaparib  Cabazitaxel | EQ-5D-5L mapped to EQ-5D-3L | The company mapped EQ-5D-5L values from PROfound to generate EQ-5D-3L values | Utility decrement on 3L: -0.023^N^ |

^A^ Outcomes were exploratory as they were not specified within the study protocol

^B^ The company applied an additional utility increment for people who received enzalutamide (+0.022) from its modelled estimate of a treatment effect of enzalutamide on quality of life from PREVAIL

^c^ Patients moved into this health state upon progression with the first line treatment (enzalutamide, abiraterone acetate, or BSC). In this health state, all patients were to receive docetaxel in 2L. This state applied to all arms of the model

^D^ Patients moved into this health state upon progression during 2L docetaxel after receiving BSC in 1L. In this health state, patients were to receive enzalutamide as a 3L treatment. This health state only applied to the BSC arm of the model

^E^ Values reported are the mean utility values, using a multi-attribute utility instrument (EQ-5D), over the last 12 months of a mHRPC patient’s life

^F^ Utility values reported were explored during the probabilistic sensitivity analysis

^G^ Utility decrements were based on 27 responses from the NHS Value in Health Panel, based on a description of a moderate disease state with and without description of most common adverse events

^H^ Utility values reported are derived from FACT-P data

^I^ Stages of early, moderate, and late advanced disease were used to describe the progression of HRPC from FACT-P data, using dimension specific scores by stage

^J^ Utility decrements from AEs and SRE

^K^ Utility decrease for progressive disease in 2L or 3L

^L^ Utility increase for patients receiving enzalutamide or abiraterone in 2L or 3L

^M^ Utility increase for patients receiving enzalutamide in 2L or 3L

^N^ From IV administration of the treatment

Abbreviations: BSC = best supportive care; EQ-5D= EuroQuol 5-Dimension; FACT-P= Functional Assessment of Cancer Therapy–Prostate; HRPC = hormone-refractory prostate cancer; HTA = health technology assessment; mCRPC = metastatic castration-resistant prostate cancer; mHRPC = metastatic hormone-refractory prostate cancer; mg = milligram; NHS = National Health Service; NICE = National Institute for Health and Care Excellence; PFS = progression-free survival; SMC = Scottish Medicines Agency; Q3W = every three weeks; QoL = quality of life; UK = United Kingdom; US = United States; VAS = visual analog scale

Table S11: EQ-5D Index Utility Values and EQ VAS from Primary Studies by Treatment

| **Treatment By Line^A^** | **Mean Baseline EQ-5D Index^B^** | | **Mean Baseline EQ VAS** | |
| --- | --- | --- | --- | --- |
|  | Lower | Upper | Lower | Upper |
| 1L Enzalutamide (n=6) | 0.71 | 0.85 | 68.0 | 77.15 |
| 1L Abiraterone acetate (n=2) | 0.87^C^ | 0.9^D^ | 70.0 | 72.8 |
| 1L Docetaxel (n=1) | 0.83^C^ | | 77.5^C^ | |
| 2L+ Enzalutamide (n=2) | 0.63 | 0.7 | 62.7 | 66.3 |
| 2L+ Abiraterone acetate (n=2) | 0.7^C^ | | 66.3 | 68.0 |
| 2L+ Cabazitaxel (n=3) | 0.699 | 0.7 | 65.8^C^ | |
| 2L+ Olaparib (n=1) | NR | NR | 65.0^C^ | |

^A^ The previous therapies received for mCRPC in the 2L+ setting were generally not reported in these studies and therefore was not considered in this summary.

^B^ Utility values from all EQ-5D instruments (i.e., EQ-5D-3L, EQ-5D-5L, unspecified EQ-5D) were combined for presentation as many studies did not detail the scale reported. Studies were reporting baseline utilities using alternative scales (such as AQoL, QWB, etc.) were not included in this results summary, but are reported in the Appendix.

^C^ Only one study reported a mean baseline utility value or VAS score for this treatment.

^D^ This study reported a median baseline EQ-5D index value as its utility value

Abbreviations: 1L = first line; 2L+ = second line and later; EQ-5D = EuroQoL 5-Dimension; n = number of studies; VAS = visual analog scale

Table S12: Utility Values by Health State from Economic Evaluations

| **1L**  **7 Studies** | | **2L+**  **12 Studies** | | **Unknown LoT**  **2 Studies** | | **Palliative/Death**  **6 Studies^A^** | |
| --- | --- | --- | --- | --- | --- | --- | --- |
| SD/PFS  6 Studies^A^ | PD  3 Studies^A^ | SD/PFS  8 Studies^A^ | PD  6 Studies^A^ | SD/PFS  2 Studies^A^ | PD  0 Studies | Palliative | Death |
| 0.63-0.844 | 0.65-0.715 | 0.617-0.83 | 0.37-0.65 | 0.5-0.85 | NA | 0.5 | 0 |

^A^ Because economic studies are not primary sources for HSUVs, it is possible that economic studies could present utility values from the same sources. To avoid repetition of the same value in this summary, each unique HSUV presented in economic studies was only included once, and the number of included studies presented in this cell represents only studies with unique HSUVs.

Note: Some economic studies did not report their HSUVs by these health state categories, were unclear which health state category the HSUV represented, or had redacted/confidential HSUVs. Therefore, those studies were left out of this results summary due to ambiguity.

Abbreviations: 1L = first line; 2L+ = second line and later; LoT = line of therapy; PD = progressed disease; PFS = progression-free survival; SD = stable disease

Table S13: Utility Values by Health State from Economic Evaluations excluding Dutch Studies

| **1L (Dutch Study Included)**  **8 Studies** | | **1L (Dutch Study Excluded)**  **7 Studies** | |
| --- | --- | --- | --- |
| SD/PFS  5 Studies^A^ | PD  3 Studies^A^ | SD/PFS  4 Studies^A^ | PD  2 Studies^A^ |
| 0.63-0.844 | 0.65-0.715 | 0.63-0.844 | 0.65-0.658 |

^A^ Because economic studies are not primary sources for HSUVs, it is possible that economic studies could present utility values from the same sources. To avoid repetition of the same value in this summary, each unique HSUV presented in economic studies was only included once.

Note: Some economic studies did not report their HSUVs by these health state categories, were unclear which health state category the HSUV represented, or had redacted/confidential HSUVs. Therefore, those studies were left out of this results summary due to ambiguity.

Abbreviations: 1L = first line; 2L+ = second line and later; PD = progressed disease; PFS = progression-free survival; SD = stable disease

Table S14: HSUVs from Economic Studies by Treatment

| **Study^A^** | **LoT** | **Utility Reported** | **Treatment** | **Value** |
| --- | --- | --- | --- | --- |
| 23-Okumura-2021 | 1L | Utility Gain | Abiraterone | 0.022 |
|  |  |  | Enzalutamide | 0.022 |
| G-HTA-470  G-HTA-471 | 2L or 3L | Utility Gain | Abiraterone or Enzalutamide | 0.04 |
| G-HTA-485  G-HTA-486  G-HTA-629 | 1L | Utility Gain | Abiraterone | 0.021 |
| G-HTA-482  G-HTA-483 | 2L | Utility of Enzalutamide after Docetaxel | Enzalutamide | 0.688 |
| 798-Collins-2007 | 1L | Disutility | Docetaxel | 0.1347 |
|  |  |  | Mitoxantrone | 0.0676 |
|  |  |  | Estramustine | 0.1097 |
| G-HTA-667 | 3L | Utility Decrement due to treatment infusion | Olaparib (infusion) | -0.023 |
|  |  |  | Cabazitaxel (infusion) | -0.023 |

^A^ Because economic studies are not primary sources for HSUVs, it is possible that economic studies could present utility values from the same sources. To avoid repetition of the same value in this summary, each unique HSUV presented in economic studies was only included once.

Abbreviations: 1L = first line; 2L = second line; 3L = third line; HTA = health technology assessment; LoT = line of therapy

Table S15: Meta-Analysis Results Summary

| **Outcome** | **n** | **Type** | **Pooled Treatment Effect Mean (95% CI)** | **Standard Deviation** | **Standard Error** |
| --- | --- | --- | --- | --- | --- |
| 1L EQ-5D-3L/5L combined – index score | 4604 | RE | 0.79 (0.75, 0.84) | 1.57 | 0.02 |
| 1L EQ-5D-5L only – index score | 1600 | RE | 0.79 (0.70, 0.87) | 1.76 | 0.04 |
| 1L EQ-5D index score – clinical trial data only | 2665 | RE | 0.83 (0.82, 0.85) | 0.40 | 0.01 |
| 1L EQ-5D index score – observational data only | 1939 | RE | 0.76 (0.70, 0.83) | 1.54 | 0.04 |
| 2L+ EQ-5D-3L/5L combined – index score | 1068 | RE | 0.69 (0.67, 0.71) | 0.27 | 0.01 |
| 2L+ EQ-5D-5L only – index score | 665 | RE | 0.68 (0.65, 0.71) | 0.40 | 0.01 |
| 2L+ EQ-5D index score – clinical trial data only | 534 | FE | 0.70 (0.69, 0.71) | 0.10 | 0.004 |
| 1L EQ VAS | 3817 | RE | 74.63 (70.97, 78.29) | 115.44 | 1.87 |
| 1L EQ VAS – clinical trial data only | 2480 | FE | 76.55 (75.88, 77.21) | 16.94 | 0.34 |
| 1L EQ VAS – observational data only | 1337 | RE | 72.62 (66.81, 78.43) | 108.36 | 2.96 |
| 2L EQ VAS | 901 | FE | 65.82 (64.53, 67.11) | 19.76 | 0.66 |
| 2L EQ VAS – clinical trial data only | 367 | FE | 66.39 (64.46, 68.33) | 18.95 | 0.99 |

Abbreviations: 1L = first line; 2L+ second line and later; CI = confidence interval; EQ-5D = EuroQoL 5-dimension; FE = fixed effects; n = sample size; RE = random effects; VAS = visual analog scale

## Supplementary Material A: Figures

Figure S1: PRISMA Flow Diagram


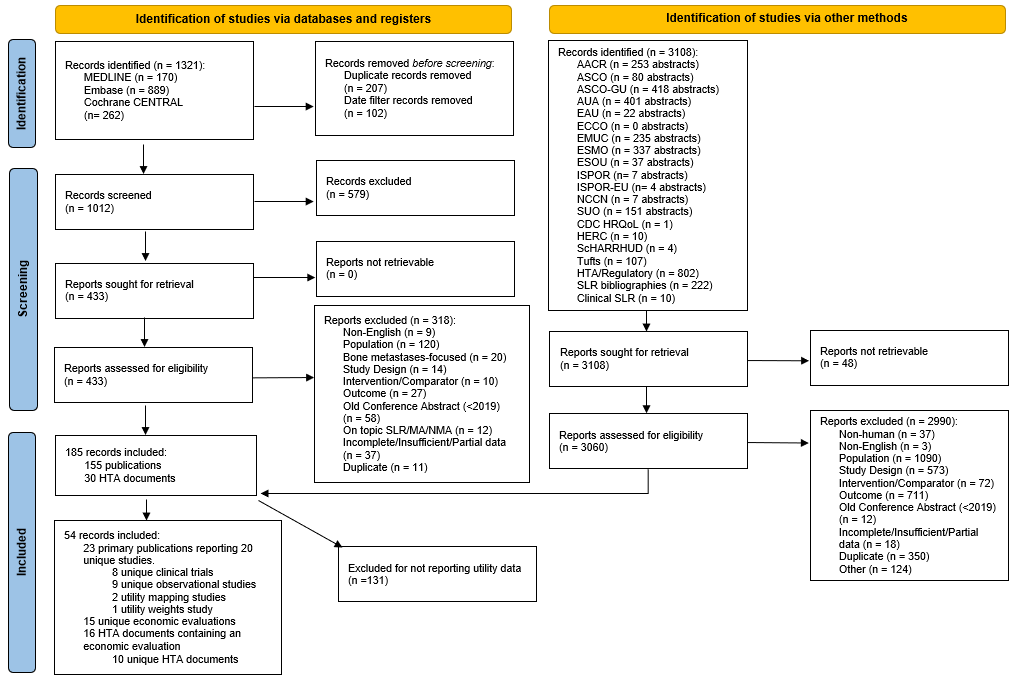


From: Page MJ, McKenzie JE, Bossuyt PM, Boutron I, Hoffmann TC, Mulrow CD, et al. The PRISMA 2020 statement: an updated guideline for reporting systematic reviews. BMJ 2021;372:n71. doi: 10.1136/bmj.n71. For more information, visit: <http://www.prisma-statement.org/>
